# Erioflorin and Erioflorin Acetate Induce Cell Death in Advanced Prostate Cancer Through ROS Increase and NF-κB Inhibition

**DOI:** 10.3390/jox15020045

**Published:** 2025-03-18

**Authors:** Cecilia Villegas, Iván González-Chavarría, Viviana Burgos, Jaime R. Cabrera-Pardo, Bernd Schmidt, Cristian Paz

**Affiliations:** 1 Laboratory of Natural Products & Drug Discovery, Center CEBIM, Department of Basic Sciences, Faculty of Medicine, Universidad de La Frontera, Temuco 4780000, Chile; c.villegas04@ufromail.cl; 2 Departamento de Fisiopatología, Facultad de Ciencias Biológicas, Universidad de Concepción, Concepción 4070386, Chile; ivancsbiologicas@gmail.com; 3 Escuela de Tecnología Médica, Facultad de Salud, Universidad Santo Tomás, Temuco 4780000, Chile; vburgos7@santotomas.cl; 4 Laboratorio de Química Aplicada y Sustentable (LabQAS), Departamento de Química, Universidad del Bío-Bío, Avenida Collao 1202, Concepción 4051381, Chile; jacabrera777@gmail.com; 5 Institut für Chemie, Universität Potsdam, Karl-Liebknecht-Str. 24-25, D-14476 Potsdam, Germany; bernd.schmidt@uni-potsdam.de

**Keywords:** *Podanthus mitiqui*, prostate cancer, erioflorin, NF-κB signaling, cytotoxicity, germacrane sesquiterpene lactones

## Abstract

Germacranes are a type of sesquiterpene lactones with anti-inflammatory and cytotoxic properties against cancer cell lines. In this in vitro study, erioflorin and erioflorin acetate were isolated and purified from the leaves of *Podanthus mitiqui* Lindl (Mitique or Mitriu), a shrub endemic to Chile and traditionally used in Mapuche medicine to treat urinary and digestive disorders. Their effects on advanced prostate cancer cell lines (DU-145 and 22Rv1) were evaluated. Cytotoxicity was assessed using real-time cell death and clonogenic assays. Apoptosis was determined by measuring reactive oxygen species (ROS), mitochondrial membrane potential (ΔΨm), and apoptotic cell percentage through flow cytometry. Gene expression of *BAX* and *BCL-2* was analyzed via RT-qPCR, while NF-κB activation was studied in DU-145 cells and human monocytic NF-κB reporter assays using LPS stimulation and alkaline phosphatase activity quantification. Erioflorin acetate exhibited the highest cytotoxicity, with IC_50_ values of 35.1 µM (22Rv1) and 27.3 µM (DU-145), compared to erioflorin, which had IC_50_ values of 50.3 µM and 56.5 µM, respectively. Both compounds increased ROS levels, reduced ΔΨm, and induced apoptosis. RT-qPCR analysis revealed that erioflorin elevated the *BAX/BCL-2* ratio, and both compounds inhibited NF-κB activation by preventing IκBα phosphorylation. In conclusion, the findings demonstrate that erioflorin and erioflorin acetate exert significant in vitro cytotoxic and cytostatic effects on prostate cancer cells, supporting their potential as natural candidates for prostate cancer therapy.

## 1. Introduction

Prostate cancer (PC) is one of the leading causes of cancer death and represents one of the leading causes of mortality worldwide. According to recent data, more than 1.4 million new cases of prostate cancer were reported in 2020, positioning it as the second most diagnosed cancer in men, after lung cancer [1]. The pathogenesis of prostate cancer is mainly driven by androgen receptor (AR) activation and aberrant nuclear factor kappa B (NF-κB) signaling, and the interaction between both pathways enhances tumor aggressiveness and therapeutic resistance [2]. Current therapies for PC include AR inhibition (chemical castration) and chemotherapy, but their efficacy is limited usually by the treatment resistance and associated side effects.

NF-κB is constitutively active in prostate cancer and plays a key role in tumor progression and relapse [3,4]. Its aberrant activation occurs in part in response to the cellular stress experienced by cancer cells. In this regard, during moderate oxidative stress, ROS induce phosphorylation and degradation of IκB, with consequent activation of NF-κB. It has been shown that phosphorylation of Ser-276 at RelA (p65) is ROS-dependent, which is required for transcriptional expression of some NF-κB target genes [5]; however, in contrast, severe oxidative stress decreases NF-κB activity, because excess ROS can interfere with the activation of IKK kinases involved upstream in the pathway [6]. NF-κB activity has been associated with castration resistance in the advanced stage of PC [7,8,9], and it shows elevated levels of nuclear p65, an indicator of NF-κB activation, compared to normal prostate tissue [3,10]. NF-κB is a target for the development of therapeutic strategies, including new natural molecules with the ability to regulate this aberrant signaling and contribute to cancer cell apoptosis, especially in advanced prostate cancer [11,12,13].

**Figure 1:**
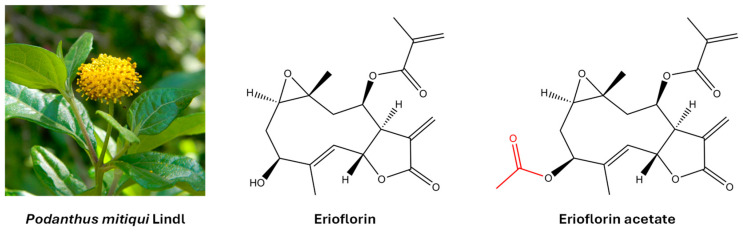
The molecular structure of the germacrane sesquiterpene lactones erioflorin and erioflorin acetate isolated from the medicinal plant *Podanthus mitiqui*.

Germacrane sesquiterpene lactones are a group of natural compounds with a ten-membered carbacycle and an annellated unsaturated lactone ring. They are widely distributed in plants of the *Asteraceae* family and are known to interact with proteins and nucleic acids, thereby modulating key cellular processes. This interaction is primarily mediated by a shared structural motif: an α,β-unsaturated γ-lactone, specifically an α-methylene-γ-lactone (also known as an α-exo-methylene-γ-lactone). This moiety exerts its biological effects by binding to nucleophilic sites of the protein (including amino, imidazole, and thiol groups) via a Michael-type reaction [14,15]. Furthermore, the presence of various polar substituents, such as epoxides, carboxylates, hydroxyls, acetoxyls, and α,β-unsaturated carbonyls—each unique to individual compounds—further influences this activity [16]. Certain germacranes induce apoptosis in cancer cells by altering cellular redox homeostasis and activating mitochondrial death [17]. In addition, they have been shown to inhibit key transcription factors in cancer, such as NF-κB and STAT3, limiting cell survival and invasion [18]. Erioflorin and erioflorin acetate were originally isolated from *Eriophyllum confertiflorum* [19] and *Podanthus ovatifolius* [20]. Later, both natural products were isolated from *Podanthus mitiqui*, a medicinal plant endemic to Chile [21,22]. Figure 1 shows their molecular structure.

Erioflorin acetate has cytotoxic activity in cell lines such as KB (human epidermoid carcinoma) and P388 (murine leukemia), in addition to low genotoxicity, suggesting an important therapeutic potential [23,24]. Erioflorin stabilizes the tumor suppressor protein Pdcd4 (Programmed Cell Death Protein 4), with the consequent transcriptional activity inhibition of Activator Protein-1 (AP-1) and NF-κB [25], which are crucial for cancer cell survival and proliferation in advanced stages of cancer. While these findings are relevant, the potential of erioflorin and erioflorin acetate in the treatment of advanced prostate cancer remains unstudied. Therefore, this study evaluated the cytotoxic and cytostatic effects of these germacranes in the DU-145 and 22Rv1 cell lines, investigating their underlying molecular mechanisms, including ROS generation, mitochondrial dysfunction, and modulation of key apoptosis proteins. Furthermore, the modulation of the NF-κB signaling pathway was analyzed, due to its pivotal role in cell survival and tumor progression in this disease.

## 2. Materials and Methods

### 2.1. Isolation and Structure Elucidation of Erioflorin and Erioflorin Acetate

Erioflorin and erioflorin acetate were isolated from the aerial parts of *Podanthus mitiqui* collected in Concepcion, VIII Region of Chile, as described previously [21,22]. Structure elucidation was accomplished by 1D and 2D-NMR spectroscopy in CD_2_Cl_2_ (CHDCl_2_ as a calibrant for ^1^H NMR spectroscopy, *δ* = 5.32 ppm; CD_2_Cl_2_ as a calibrant for ^13^C NMR spectroscopy, *δ* = 53.8 ppm). All NMR spectra were recorded at 500 MHz (^1^H NMR spectroscopy) and 125 MHz (^13^C NMR spectroscopy), respectively, using a Bruker Avance NEO 500 spectrometer (BrukerBiospin GmbH, Rheinstetten, Germany). Signal assignments are based on the 2D-NMR experiments H,H-COSY, NOESY, HSQC, and HMBC. Copies of all spectra, full signal assignments, and comparison with reference data available in the literature are provided in the Supporting Information. Copies of NMR spectra for erioflorin are provided in Figures S1–S6 and for erioflorin acetate in Figures S7–S11.

### 2.2. Cell Culture

Human prostate epithelial cells, DU-145 and 22Rv1, were obtained from the American Type Culture Collection (ATCC) and cultured in RPMI-1640 medium (Cytiva) supplemented with 10% Fetal bovine serum (FBS) (Cytiva), 100 U/mL penicillin, and 0.1 mg/mL streptomycin (Cytiva) at 37 °C in a humidified atmosphere with 5% CO_2_. DU-145 cells are derived from a metastatic brain tumor of a prostate cancer patient and correspond to advanced prostate cancer expressing functional receptor NF-κB activator, whereas 22Rv1 cells are derived from a human prostate carcinoma xenograft (CWR22), express the androgen receptor and prostate-specific antigen, and are androgen-independent.

### 2.3. IncuCyte^®^ Real-Time Cell Death Assay

The real-time cytotoxicity of erioflorin and erioflorin acetate was evaluated. DU-145 and 22Rv1 cells (20,000 cells/well) were seeded in 96-well plates and treated with increasing concentrations of erioflorin and erioflorin acetate (6–200 μM) in RPMI-1640 medium containing 0.1% (*v*/*v*) DMSO. The experiments were conducted using the IncuCyte^®^ S3 live-cell analysis system (Bohemia, NY, USA), with 30 nM Sytox Green dye (Invitrogen™, #S7020, Carlsbad, CA, USA) as a cell death marker [26,27]. Dead cells were then automatically counted every hour for 48 h. Cell death was analyzed based on green fluorescent and IC_50_ values of each molecule were calculated from the data generated for each condition assayed using Graphpad Prism 8.0 software. The confluence area of the monolayer defined as the percentage of the image area that is occupied by objects was calculated using IncuCyte^®^ v2019B software from the Advanced Microscopy Center (CMA Biobio, Concepción, Chile) of the University of Concepción.

### 2.4. Selection of the Standard Working Concentration

The working concentration for erioflorin and erioflorin acetate was set at 50 μM for the 24 h experiments. At this concentration, cell death is almost 50%, and using the same concentration for both compounds allows for a comparison of their effects.

### 2.5. Clonogenic Assays

Determination of the antiproliferative activity of erioflorin and erioflorin acetate at concentrations below the IC_50_ value was confirmed by a clonogenic assay; cells were grown at a density of 1000 per well in a 12-well plate and then incubated with increasing concentrations of erioflorin and erioflorin acetate (0–25 µM) in RPMI-1640 medium. After 6 h, the compound was removed, and the cells were cultured for 14 days with refreshed RPMI-1640 medium. Colonies were fixed with 80% ethanol for 15 min at room temperature, stained with 0.5% crystal violet for 20 min, and photographed [28]. The total colony area was calculated for each condition using Image J software V.1.49 (NIH, Bethesda, MD, USA) [29].

### 2.6. Cell Apoptosis Detection by Flow Cytometry

Cell apoptosis was assessed using the Annexin V-Alexa Fluor™ 488 Apoptosis Detection Kit with propidium iodide (cat#V13241, Invitrogen™) following the method proposed by Lakshmanan and Batra as a reference [30]. DU-145 and 22Rv1 cells were seeded at a density of 5 × 10^5^ cells/mL in 60 mm petri dishes and cultured for 24 h. Subsequently, cells were treated with 50 µM erioflorin or erioflorin acetate, 0.4 µM camptothecin (positive control), or 0.1% DMSO (negative control) for an additional 24 h. Following treatment, cells were trypsinized, harvested, and stained with Annexin V/PI at room temperature for 15 min in the dark. Stained cells were analyzed using a Becton Dickinson FACSCanto II flow cytometer with excitation at 488 nm and emission at 530 nm. Cell populations were quantified based on their staining profiles: early apoptotic cells (Annexin V-positive, PI-negative), late apoptotic/necrotic cells (Annexin V-positive, PI-positive), and necrotic cells (Annexin V-negative, PI-positive).

### 2.7. ROS Measurement

Cells were seeded at densities of 15,000 cells per well in a black flat-bottomed 96-well plate and incubated at 37 °C with 5% CO_2_ in RPMI-1640 culture medium without phenol red. After 24 h, the cells were incubated with 5 μM of 2′,7′-dichlorodihydrofluorescein diacetate (H2DCFDA) (#5935, Tocris Bioscience, Bristol, UK) for 30 min in accordance with the manufacturer’s protocol and taking as a reference the description in [31], and then treated with 50 μM erioflorin and erioflorin acetate. DMSO 0.1% was used as control. Fluorescence was then recorded every hour for 20 h using the VICTOR^®^ Nivo™ microplate reader at ex 495 nm/em 520 nm.

### 2.8. Mitochondrial Membrane Potential Analysis

This analysis was performed in real-time using the IncuCyte^®^ analysis system, with Image-iT^®^ TMRM reagent (Invitrogen™, #I34361) as a mitochondrial membrane potential indicator, adjusting the manufacturer’s instructions to the protocol recommended by [32]. DU-145 and 22Rv1 cells (20,000 cells/well) were seeded in 96-well plates for 24 h. TMRM stain (100 nM) was then added, and the cells were incubated for 30 min. Afterward, cells were treated with 50 μM erioflorin, erioflorin acetate, or vehicle (DMSO 0.1%). Plates were kept in incubation and fluorescence intensity was recorded every hour for 24 h. Images were processed using IncuCyte^®^ software (v.2019B).

### 2.9. Quantitative Real-Time PCR Analysis

22Rv1 cells were cultured and treated with 50 μM erioflorin, erioflorin acetate, or DMSO 0.1% for 12 h. Total RNA was isolated using RNA-Solv Reagent (Omega Biotek, Inc., Norcross, GA, USA). A total of 1000 ng of total RNA was reverse transcribed (RT) to obtain cDNA using a High-Capacity cDNA Reverse Transcription Kit (Applied Biosciences, Foster City, CA, USA) according to the manufacturer’s protocol. The cDNA was used to determine expression levels of the housekeeping Ribosomal Protein L27 (RPL27) gene (Forward 5′-TCCGGACGCAAAGCTGTCATC-3′, reverse 5′-GGTCAATTCCAGCCACCAGAGCAT-3′) [33] and genes of interest (BAX and BCL-2). Standard curves were performed using serial dilutions of specific BAX (Forward 5′-ATGTTTTCTGACGGCAACTTC-3′, reverse 5′-AGTCCAATGTCCAGCCCAT-3′) [34], BCL-2 (Forward 5′-GGATAACGGAGGCTGGGATG-3′, reverse 5′-GGGCCAAACTGAGCAGAGTC-3′) [35,36], and RPL27 genes with a Brilliant SYBR^®^ Green qPCR Master Mix kit, (cat#600548 Agilent Technologies, Santa Clara, CA, USA). Specific primers were obtained from Integrated DNA Technologies (Coralville, IA, USA) and qPCRs were performed on a Bioneer Real-Time Quantitative PCR system (Exicycler™ 96) for 40 cycles. Results were analyzed as relative 2-ΔΔCT quantification.

### 2.10. Analysis of NF-κB Activation

NF-κB activation was evaluated by an alkaline phosphatase reporter assay in human monocytic THP1-Blue™ cells (InvivoGen, San Diego, CA, USA) stably transfected with a secreted embryonic alkaline phosphatase gene (SEAP) that is under the control of an NF-κB-inducible promoter. THP1-Blue cells (100,000 cells/well) were pretreated with erioflorin and erioflorin acetate or DMSO 0.1% for 30 min, followed by an addition of 100 ng/mL LPS for 24 h [37]. Alkaline phosphatase activity was determined in cell supernatants using a QUANTI-Blue mixture (InvivoGen) according to the manufacturer’s specifications. Absorbance was measured at 655 nm and compared with positive control samples (LPS). Subsequently, the percentage of inhibition was calculated, and the difference was contracted with the sample stimulated only with LPS, which represented 100% of the activation of the pathway.

### 2.11. Western Blot Analysis

DU-145 cells (1 × 10^6^ cells/well) were seeded and treated with 50 µM erioflorin and erioflorin acetate with and without LPS (1 µg/mL). After 1 h incubation, cells were washed twice with precooled PBS. The cells were lysed with RIPA Lysis Buffer System^®^ (#sc-24948, Santa Cruz Biotechnology, Inc., Dallas, TX, USA) containing protease and phosphatase inhibitors. The cells were scraped and transferred to 1.5 mL tubes, and centrifuged at 12,000× *g* for 20 min. The supernatant was collected, and the protein concentration was measured using a Pierce™ BCA protein assay kit (#23227, Thermo Scientific™, Waltham, MA, USA). Subsequently, 30 μg of proteins were separated by electrophoresis and transferred to a PVDF (polyvinylidene fluoride) membrane. The membrane was incubated overnight with the primary antibody in Tris-buffered saline with Tween20^®^ at 4 °C. Antibodies against IκBα (1:1000, #4812, Cell signaling Technology, Danvers, MA, USA), Phospho-IκBα (P-IκBα), (1:1000, #9246, Cell signaling Technology) and α-tubulin (1:1000, #sc-5286, Santa Cruz Biotechnology, Inc.) were used. Immunoreactive bands were visualized using a horseradish peroxidase-conjugated secondary antibody (Anti-Mouse IgG, #715-035-150; Anti-Rabbit IgG 711-035-152, 1:10,000, Jackson Immunoresearch Inc., West Grove, PA, USA) and chemiluminescence detection with chemiluminescent substrate SuperSignal™ West Pico PLUS (#34579, Thermo Scientific™). Images were obtained with a G:Box chemi XRQ gel doc system (Syngene) and band densitometry analysis was performed with Image J software V.1.49 (NIH, Bethesda, MD, USA).

## 3. Results

### 3.1. Chemical Characterization of Purified Compounds from Podanthus mitiqui

The aerial parts of *Podanthus mitiqui* were powdered and macerated with ethyl acetate (EtOAc) for 3 days, yielding 250 g of crude extract. This extract was fractionated by column chromatography (silica gel 60/70–210 mesh) into 11 fractions (F1–F11) by gradually increasing the polarity of the eluent. Fraction F-8, which contained the highest amount of erioflorin, was further purified, resulting in two white solids with different polarities. These solid products were recrystallized from EtOAc, producing colorless crystals suitable for X-ray diffraction and NMR analysis (>99% purity). These analyses confirmed the erioflorin structure, consisting of a central ten-membered carbon ring with two annellated heterocycles (an epoxide between C1 and C10 and a butyrolactone between C6 and C7) and a five-carbon unsaturated ester side chain attached to C8. The main structural difference between erioflorin and its acetate derivative was the presence of an acetyl group at C3 (See Figure 1). NMR data assignments are summarized in the Supplementary Materials.

### 3.2. Erioflorin and Erioflorin Acetate Show Cytotoxic Effects Against DU-145 and 22Rv1 Cells Lines

**Figure 2:**
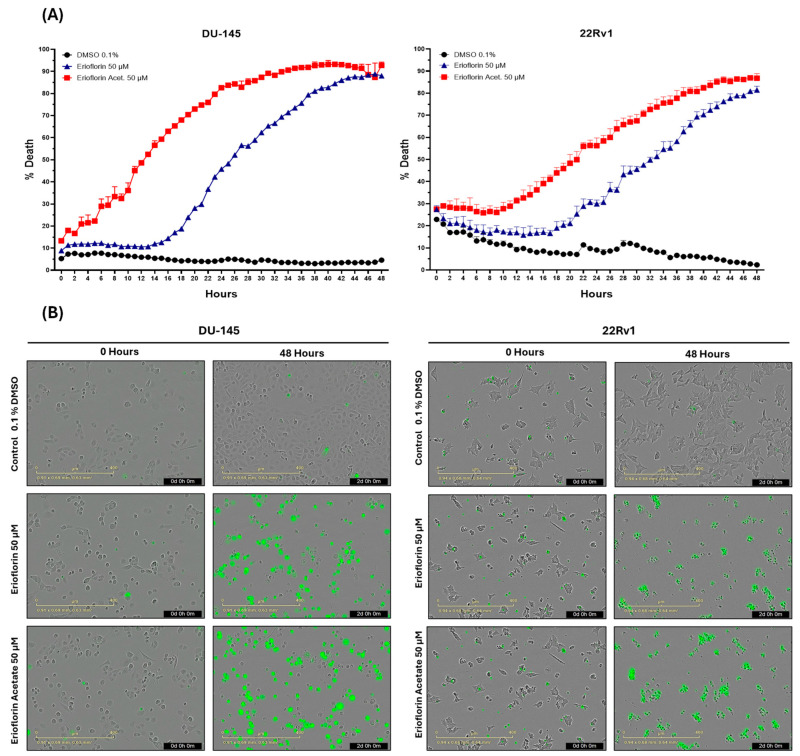
The IncuCyte^®^ live-cell analysis system used to study the effect of treatment with the germacranes erioflorin and erioflorin acetate on cell death in DU-145 and 22Rv1 cell lines. (**A**) Real-time plots for cell death measurements (48 h, %) in cells exposed to 50 μM of germacranes. Data are presented as mean ± SD (n = 3). (**B**) Microscopy images of Sytox Green-stained cells in DU-145 and 22Rv1 cells treated with erioflorin and erioflorin acetate (50 μM), observations at 0 and 48 h at the end of treatment.

The real-time cytotoxicity assessment using the IncuCyte^®^ live-cell analysis system, as shown in Figure 2A, indicated that both germacranes are cytotoxic against advanced PC cell lines. However, erioflorin acetate exhibited higher potency than erioflorin against DU-145 and 22Rv1 cells at 48 h. Additionally, DU-145 cells were more sensitive, showing an earlier onset of cell death compared to 22Rv1 cells.

Microscopy images obtained for each hour during the whole experiment revealed that treatment with erioflorin and erioflorin acetate (50 μM) induced a progressive increase in Sytox signal (green), indicating enhanced plasma membrane permeability, a characteristic marker of cell death. These effects were observed in both the DU-145 and 22Rv1 cell lines after 12 h of incubation and became more evident after 48 h of treatment (Figure 2B). Morphological analysis showed characteristic apoptotic changes, including cell shrinkage, apoptotic body formation, and loss of monolayer integrity, particularly in the DU-145 cell line, where a higher proportion of Sytox-positive cells was observed compared to the 22Rv1 cell line. In some cells, a prominent swelling of the membrane was observed, which could be indicative of necrosis or a late-stage apoptotic event.

**Figure 3:**
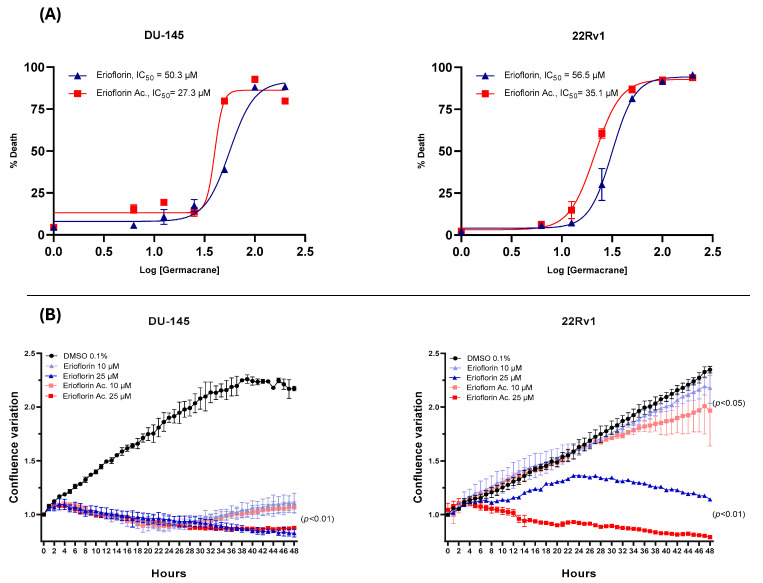
Dose–response plots (6–200 μM) for erioflorin and erioflorin acetate showing percent cell death, and real-time measurements of monolayer growth relative to the control. (**A**) Dose–response curves evaluated using IncuCyte^®^ at 48 h. The percentage of cell death is plotted as a function of the logarithmic concentration of the compounds, with the IC_50_ value for each compound indicated on the graph. (**B**) Time–course analysis of cell confluence in DU-145 and 22Rv1 cell lines treated with erioflorin and erioflorin acetate at 0, 10, and 25 μM. Data are presented as mean ± SD (n = 3).

The dose–response curve plots displayed in Figure 3A show that erioflorin acetate exhibited the most potent cytotoxic activity, with IC_50_ values of 35.1 µM and 27.3 µM against 22Rv1 and DU-145, respectively, while erioflorin showed IC_50_ values of 50.3 µM and 56.5 µM against the same cells. In addition, the real-time confluence measurements by IncuCyte^®^ (Figure 3B) showed that erioflorin and erioflorin acetate inhibit proliferation at non-toxic concentrations (25 µM) after 24 h compared to the untreated control (*p* < 0.05). In this regard, it was observed that the cytostatic effects are more pronounced in DU-145 cells, with both germacranes arresting cell proliferation at 10 µM.

### 3.3. Erioflorin and Erioflorin Acetate Reduce the Proliferative Activity of DU-145 and 22Rv1cells

**Figure 4:**
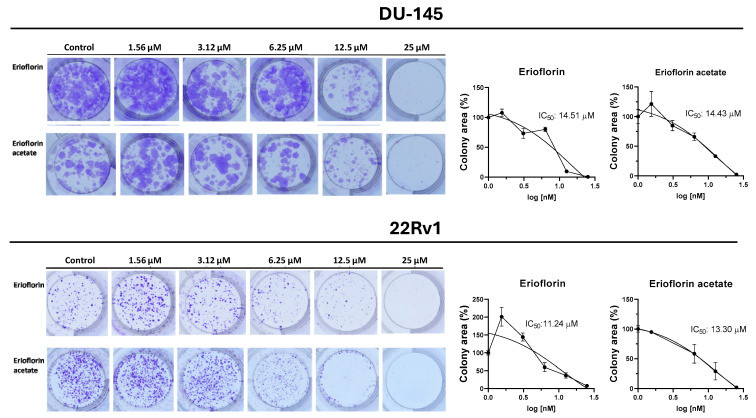
Colony formation assays on DU-145 and 22Rv1 cell lines exposed to erioflorin and erioflorin acetate at non-cytotoxic concentrations (1.56–25 µM) for 3 h (**Left panel**): Representative images of colonies stained with crystal violet after 11 days of culture following germacrane exposure (**Right panel**): IC_50_ values for colony formation were determined. The percentage of colony area was quantified using ImageJ software V.1.49, enabling precise analysis of treatment effects on long-term cell viability and proliferation.

Germacrane sesquiterpenoids have been reported to exhibit cytostatic and pro-apoptotic effects, including the induction of cell cycle arrest in PC cells [38,39]. Given that the germacranes evaluated in this study inhibited cell proliferation at 48 h, it was investigated whether prolonged exposure would prevent colony formation in advanced prostate cancer cells. Figure 4 shows the results of the 11-day clonogenic assays, evidencing that erioflorin and erioflorin acetate effectively inhibited colony formation in the DU-145 and 22Rv1 cell lines. The IC_50_ values for erioflorin were 14.51 µM in DU-145 and 11.24 µM in 22Rv1, while for erioflorin acetate they were 14.43 µM and 13.30 µM, respectively.

### 3.4. Erioflorin and Erioflorin Acetate Have Pro-Apoptotic Effects on DU-145 and 22Rv1 Cells

**Figure 5:**
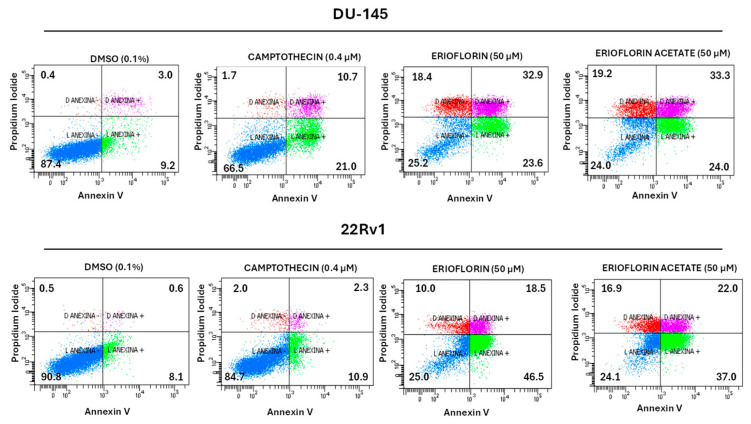
Flow cytometry analysis of cell death induction by erioflorin and erioflorin acetate (50 µM) in DU-145 and 22Rv1 prostate cancer cells. Annexin V and propidium iodide (PI) were used to classify cells as viable (Annexin V−/PI−), early apoptotic (Annexin V+/PI−), late apoptotic or secondary necrotic (Annexin V+/PI+), and primary necrotic (Annexin V−/PI+). Camptothecin (0.4 µM) was used as a positive control and DMSO (0.1%) as a negative control. Both compounds significantly reduced cell viability and increased apoptosis, with stronger effects in 22Rv1 cells. In each quadrant, the % of the corresponding cell states is shown.

To elucidate the mechanism underlying the cytotoxic and cytostatic activity exhibited by erioflorin and its acetate, an apoptosis assay was performed using Annexin V and propidium iodide (PI), which allowed us to distinguish between viable, apoptotic, and necrotic cells. Flow cytometry apoptosis assay showed that both erioflorin and erioflorin acetate significantly induce apoptosis at 50 µM after 24 h incubation, like camptothecin, which was used as a positive control due to its well-documented ability to reliably induce apoptosis in a wide variety of cell lines via topoisomerase I (Figure 5). In DU-145 cells, erioflorin (50 µM) induced a decrease of 25.2% in viable cells, with an increase in late apoptosis (32.9%) and mild early apoptosis (23.6%). Erioflorin acetate has a similar profile. In 22Rv1 cells, erioflorin at 50 µM led to a decrease in viable cells (25%) with a marked increase in early apoptosis (46.5%) and moderate late apoptosis (18.5%). Erioflorin acetate similarly reduced viability (24.1%), with late (22.0%) and early apoptosis (37.0%). However, compared with camptothecin, in both cell lines, the germacranes induced primary necrosis in a major proportion of cells (Figure 5). These results suggest that both compounds are cytotoxic through a combination of different types of cell death.

### 3.5. Erioflorin and Erioflorin Acetate Increase Cellular ROS Production and Decrease Mitochondrial Membrane Potential

**Figure 6:**
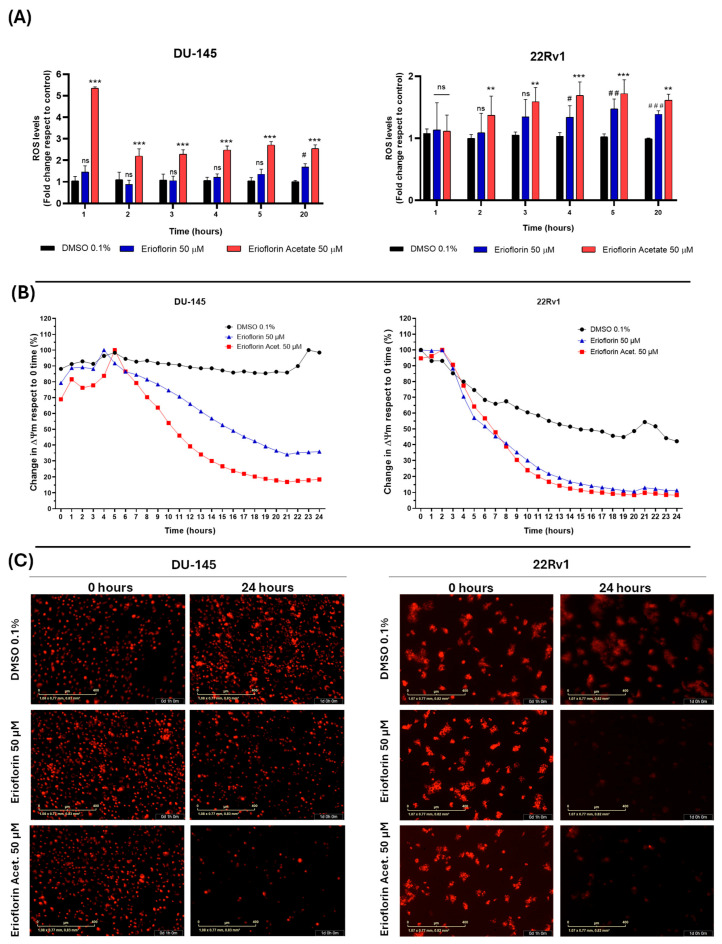
Effects of the germacranes erioflorin and erioflorin acetate on cellular ROS production and ΔΨm in DU-145 and 22Rv1 cell lines. (**A**) Quantification of ROS production using the fluorescent probe H_2_DCFDA. Significant differences between erioflorin and the control are marked with # (# *p* < 0.05, ## *p* < 0.01, ### *p* < 0.001), while differences between erioflorin acetate and the control are indicated with * (* *p* < 0.05, ** *p* < 0.01, *** *p* < 0.001), no significant differences are indicated as “ns”. (**B**) Time-dependent reduction in ΔΨm following treatment with germacranes (50 μM), measured by the decline in TMRM fluorescence intensity relative to the control. Data are presented as the means of three replicates. (**C**) Representative microscopy images of TMRM fluorescence at time 0 and the end of the experiment (24 h). The marked decrease in red fluorescence corresponds to ΔΨm dissipation, as TMRM diffuses upon mitochondrial depolarization.

The results demonstrated that erioflorin acetate significantly increased cellular ROS generation, exhibiting a more potent effect, although with slightly different behavior of the two cell lines. Specifically, in DU-145 cells, ROS levels increased abruptly within the first hour of treatment, followed by stabilization, possibly due to the activation of endogenous antioxidant mechanisms. In contrast, 22Rv1 cells exhibited a progressive increase in ROS generation throughout the incubation period. Erioflorin also significantly increased ROS production, although this effect was observed later than with erioflorin acetate (Figure 6A). Regarding the impact of germacranes on mitochondrial function, kinetic studies of depolarization using IncuCyte^®^ live-cell time-course analysis revealed a significant decrease in ΔΨm in both DU-145 and 22Rv1 cell lines compared to the control, beginning at 6 h of incubation (Figure 6B). These findings were further corroborated by microscopic images acquired at baseline (time 0) and at 24 h, which showed a marked decrease in red fluorescence, indicative of ΔΨm loss (Figure 6C).

**Figure 7:**
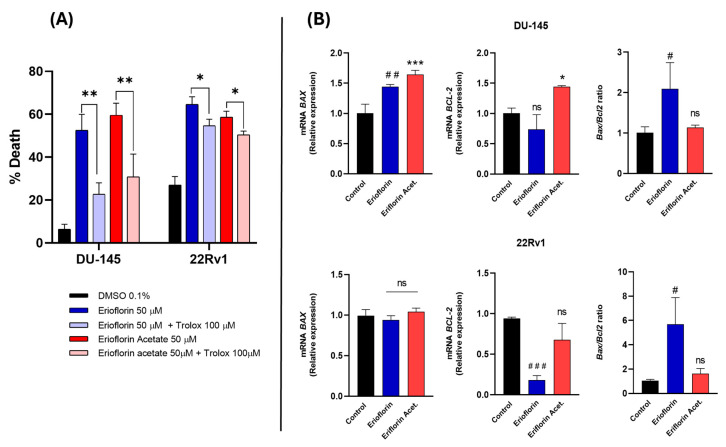
(**A**) Effect of Trolox on erioflorin- and erioflorin acetate-induced cell death (%) in DU-145 and 22Rv1 cells after 24 h. Significant differences between the compound alone or in the presence of Trolox are indicated with * (* *p* < 0.05, ** *p* < 0.01), no significant differences are indicated as “ns”. (**B**) Quantitative real-time PCR analysis of *BAX* and *BCL-2* gene expression in DU-145 and 22Rv1 cell lines treated with erioflorin and erioflorin acetate. Expression levels of *BAX* (proapoptotic) and *BCL-2* (antiapoptotic) were normalized to untreated controls and expressed as fold change. Data are represented as mean ± SD of three independent experiments. Significant differences between erioflorin and the control are marked with # (# *p* < 0.05, ## *p* < 0.01, ### *p* < 0.001), while differences between erioflorin acetate and the control are indicated with * (* *p* < 0.05, *** *p* < 0.001), no significant differences are indicated as “ns”.

To confirm whether the cytotoxicity of the compounds was directly associated with ROS production, DU-145 and 22RV1 cells were subsequently incubated with 50 µM erioflorin and erioflorin acetate in the presence/absence of Trolox (100 µM), a vitamin D analog and a potent ROS inhibitor, using Sytox Green dye as a cell death marker, and the change in fluorescence intensity at time 0 and 24 h incubation was recorded. Trolox significantly inhibited cell death induced by both erioflorin and its acetate (Figure 7A), confirming that ROS play a direct role in mediating the cytotoxic effects of these compounds.

### 3.6. Erioflorin Increases BAX/BCL-2 Ratio in Advanced PC Cell Lines

BAX and BCL-2 are key proteins in the regulation of mitochondrial apoptosis, and it is known that natural germacrane-like compounds can negatively regulate anti-apoptotic proteins and promote the expression of pro-apoptotic proteins such as BAX, reversing the balance towards apoptosis. Real-time PCR was employed to analyze the BAX/BCL-2 mRNA expression ratio in DU-145 and 22Rv1 cells after 8 h of treatment with 50 μM erioflorin or erioflorin acetate. The results of this analysis are shown in Figure 7B. Erioflorin significantly increases the BAX/BCL-2 ratio by 3.2-fold and 1.5-fold in 22Rv1 and DU-145, respectively. In contrast, erioflorin acetate did not show this imbalance, or at least without any statistical significance.

### 3.7. Erioflorin and Erioflorin Acetate Inhibit IκBα Phosphorylation

**Figure 8:**
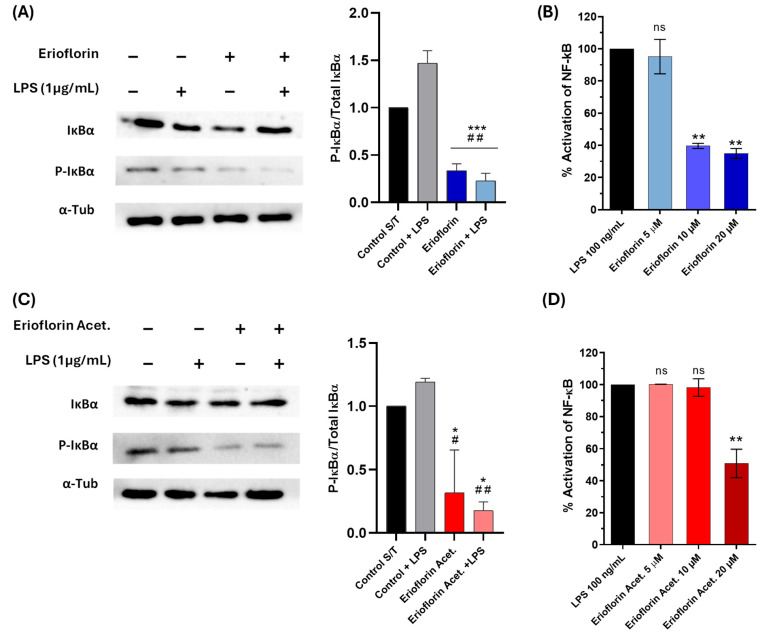
Effect of the germacranes erioflorin and erioflorin acetate on the NF-κB pathway. (**A**,**C**): Western blot analysis of IκBα and phospho-IκBα in DU-145 cells treated with erioflorin and erioflorin acetate, with or without LPS for 1 h. The graph shows the ratio of phospho-IκBα to IκBα. α-Tubulin was used as a loading control. Significant differences between compounds and the control are indicated with * (* *p* < 0.05, *** *p* < 0.001), while differences between compounds and the control + LPS are indicated with # (# *p* < 0.05, ## *p* < 0.01). (**B**,**D**): NF-κB responses in THP1- Blue™ NF-κB cells expressed as percentages. Cells were incubated with LPS for 24 h. After incubation, NF-κB-induced SEAP activity was assessed. Differences between the compounds and LPS are indicated with * (** *p* < 0.01), no significant differences are indicated as “ns”. Data are shown as percentages based on OD at 650 nm (mean ± SD).

Bless et al. reported that erioflorin inhibits the activity of tumor-associated transcription factors regulated by Pdcd4 and IκBα, such as AP-1 and NF-κB, which alters cell cycle progression and suppresses proliferation of various cell lines [25]. In this study, the inhibition of phosphorylated IκBα (P-IκBα) was assessed in DU-145 by Western blot analysis. IκBα phosphorylation served as a marker of NF-κB pathway activation. Under LPS stimulation, elevated phosphorylation of IκBα was observed, in contrast to the levels observed after treatment with erioflorin and erioflorin acetate at 50 µM, which showed a significant reduction in phosphorylated IκBα compared to the control (LPS) (Figure 8A,C), suggesting that both compounds inhibit the NF-κB pathway by preventing IκBα phosphorylation in DU-145 cells, possibly by stabilizing non-phosphorylated IκBα, thus preventing p50/p65 dimer release.

### 3.8. Germacranes Reduce the Activity of the NF-κB Pathway in the THP-1 Reporter Cell at Non-Cytotoxic Concentrations

To confirm the previous result, the inhibition of the NF-κB signaling pathway was evaluated in THP-1 reporter cells exposed to the compounds erioflorin and erioflorin acetate at concentrations of 5, 10, and 20 µM, using SEAP production as an activity marker. Stimulation with LPS activated the NF-κB pathway, reaching 100% SEAP production. For erioflorin, SEAP production showed a significant decrease starting at 10 µM, reaching an approximately 61% reduction (*p* < 0.001), while at 20 µM, activation was reduced to 65% (*p* = 0.001), indicating a dose-dependent inhibition (Figure 8B). In the case of erioflorin acetate, a similar trend was observed, though with a lesser effect at lower concentrations. Significant inhibition of phosphatase alkaline began at 20 µM, reducing NF-κB activation to approximately 40% (*p* < 0.05) (Figure 8D). Neither of the germacranes had any effect at 5 µM. These results suggest that both compounds inhibit the NF-κB pathway in a concentration-dependent manner, with erioflorin being slightly more effective overall than its acetate derivative in reducing SEAP production.

## 4. Discussion

The activities of erioflorin acetate and erioflorin methacrylate were previously studied by Cea et al., showing that these compounds have low genotoxicity in mouse bone marrow cells [24]. Subsequently, Blees and collaborators found that erioflorin inhibits the proliferation of MCF7, HeLa, and RKO cells within 2.5 and 5 µM, for 6 days, not affecting the viability in HEK293 (non-cancerous cells) [25].

In this study, the bioactive compounds erioflorin and erioflorin acetate from *Podanthus mitiqui* were evaluated, revealing that both exhibit cytotoxicity against DU-145 and 22Rv1 advanced prostate cancer cell lines, with IC_50_ values of 56.5 and 50.3 μM, respectively, while its ester, erioflorin acetate, is more potent, with IC_50_ values of 27.3 μM and 35.1 μM (Figure 3), indicating the relevance of the hydroxyl group at the C3 position of the central ring (Figure 1). DU-145 is a cell line characterized by no expression of AR and is more associated with a resistant cancer stage. Moreover, at concentrations lower than IC_50_, both compounds displayed antiproliferative activity over 48 h by IncuCyte^®^ (Figure 3B), with higher sensitivity in 22Rv1 cells. Also, the clonogenic assays showed this effect at concentrations around 15 µM for 11 days post-treatment (Figure 4). These results support the potential of these sesquiterpenes as cytotoxic and cytostatic agents and open new perspectives for their development as antitumor drugs. Other germacranes, like costunolide, inhibit proliferation in both hormone-dependent (LnCaP) and hormone-independent (DU-145 and PC-3) prostate cancer cells, with effective concentrations in the range of 3–5.9 µM [38,39]. The application of the germacrane K100 and parthenolide in MDA-MB-231 triple-negative breast cancer cells prolong the S phase without affecting other phases of the cell cycle, highlighting their ability to delay cell division without inducing toxicity at low concentrations.

Real-time cytotoxicity analysis using IncuCyte^®^ demonstrated a progressive increase in the fluorescence signal, corresponding to cell death after loss of plasma membrane continuity [26]. This process is more rapid with erioflorin acetate compared to erioflorin (Figure 2). Morphological changes indicative of apoptosis, such as cell shrinkage and chromatin condensation, were observed [40]. Subsequently, some cells exhibited swelling of their plasma membrane (PM) prior to the emission of the green fluorescent signal (Figure 2B). This loss of PM continuity, referred to as secondary necrosis [41,42], is morphologically characterized by swelling and lysis. It is important to note that ATP depletion in apoptotic cells can lead to an oncotic process, representing an intermediary stage between apoptosis and secondary necrosis [43]. Quadrant analysis in the annexin V/PI apoptosis assay by flow cytometry demonstrated that both erioflorin and erioflorin acetate significantly increased the proportion of Annexin V+/PI− and Annexin V+/PI+ cells, similar to the effect observed with camptothecin, a known apoptosis inducer (Figure 5). This suggests that erioflorin and erioflorin acetate induce both early and late apoptosis. However, erioflorin acetate resulted in a higher proportion of secondary necrosis (Annexin V+/PI+) and primary necrosis (Annexin V−/PI+), which occurs without prior phosphatidylserine exposure. This suggests that these compounds may not be exclusively specific to the induction of apoptosis. This implies that these compounds may also influence other regulated cell death processes, such as ferroptosis or necroptosis, which also involve phosphatidylserine exposure [42,44].

In addition, the mechanisms underlying the cytotoxic activity of these molecules appear to involve multiple pathways. In the first stage, both compounds induce a progressive increase in overall ROS production, but erioflorin acetate does this faster than erioflorin during the first two hours of treatment (Figure 6A). Then, a decrease in mitochondrial membrane potential was evident, which occurred abruptly after 8 h of treatment (Figure 6B). ROS accumulation beyond the capacity of antioxidant defenses is known to directly damage DNA, proteins, and lipids, leading to mitochondrial membrane permeabilization and the initiation of cell death cascade [45,46,47]. The findings of this study confirm that ROS generation is crucial for germacrane-induced cell death, as co-administration of Trolox effectively prevented cell death induced by erioflorin or erioflorin acetate by preventing oxidative damage (Figure 7A). Similar behavior has been observed with other germacranes at comparable concentrations. For instance, parthenolide induces apoptosis in HT-29 cells treated with 40 µM, which is closely associated with the loss of ΔΨm [48]. Likewise, carpescernolide exhibits dose-dependent cytotoxic effects in SMMC-7721 cells (hepatocellular carcinoma) with an IC_50_ of 38.86 µM, demonstrating a significant increase in ROS levels in treated cells [49]. However, the germacrane deoxyelephantopine showed an improved IC_50_ of between 2.9 and 5.8 µM and was found to induce apoptosis mediated by endoplasmic reticulum stress and the JNK pathway in ROS-triggered mammary carcinoma cells [50].

Mitochondrial dysfunction-mediated apoptosis is a process associated with an imbalance between pro-apoptotic and anti-apoptotic proteins [51]. Based on our findings, erioflorin significantly increased the BAX/BCL-2 expression ratio in both cell lines, although in different magnitudes (3.8-fold in 22Rv1 and 1.3-fold in DU-145), suggesting a more pronounced pro-apoptotic effect in 22Rv1 cells (Figure 7). In contrast, erioflorin acetate did not alter the BAX/BCL-2 ratio in both cells, which may indicate that acetylation reduces the apoptotic pathway. Although this compound displayed higher toxicity and antiproliferative activity, this could be caused by ROS increasing over a specific pathway.

Upon analyzing the effect of erioflorin on BAX mRNA expression in the DU-145 cell line, a significant increase was observed. However, no significant effect on BCL-2 mRNA expression was detected (Figure 7A). Therefore, it is inferred that, in the DU-145 line, the increase in BAX expression induced by erioflorin was sufficient to trigger apoptosis, regardless of BCL-2 levels. This is consistent with BAX being a key effector in mitochondrial membrane permeabilization, both in vitro and in vivo [52]. However, in the 22Rv1 cell line, the increase in the BAX/BCL-2 ratio occurred at the expense of a decrease in BCL-2 expression, sensitizing the cell to mitochondrial death (Figure 7B). In this regard, studies have shown that, even without a significant increase in BAX, the reduction in BCL-2 expression can be sufficient to destabilize the mitochondrial membrane potential, especially in cancer cells dependent on BCL-2 for survival. This is because increased expression of these anti-apoptotic proteins can suppress apoptosis by preventing the activation of BAX and BAK [53,54]. In this context, one study demonstrated that 22Rv1 cells exhibit nearly undetectable BAX protein levels and that blocking Akt, which targets the BCL-2 signaling pathway, sensitizes castration-resistant prostate cancer cells to enzalutamide [53]. These observations highlight the role of erioflorin in a cellular context where BCL-2 is predominant, and BAX is limited, suggesting it may be more effective in cells with high basal BCL-2 levels [55].

To date, erioflorin activity has been mainly attributed to its ability to stabilize the tumor suppressor protein Pdcd4 [25], a tumor suppressor that inhibits protein translation and activation of the mTORC2-Akt axis, which induces apoptosis and arrests cell growth [56]. Erioflorin has also been identified as an inhibitor of βTrCP, a ubiquitin ligase involved in the degradation of IκBα, an inhibitor of the transcription factor NF-κB [25]. Therefore, degradation of IκBα facilitates NF-κB activation and vice versa. In this context, the ability of the compounds erioflorin and erioflorin acetate to inhibit the NF-κB signaling pathway, which is overexpressed in DU-145 cancer cells and plays a pivotal role in carcinogenesis, was evaluated [57]. NF-κB/p65 is constitutively activated in human prostate adenocarcinoma and this is associated with tumor progression to advanced stages [58]. Erioflorin and erioflorin acetate prevented the canonical NF-κB activation in DU-145, showing inhibitory activity on the phosphorylation of IκBα (Figure 8A,C); however, erioflorin reduced SEAP production by 61% at 10 µM (*p* < 0.01), whereas erioflorin acetate shows this effect above 20 µM in the THP-1 reporter cell (Figure 8B,D). These findings are congruent with previous studies that reported antitumor activity by germacrane sesquiterpene lactones that act predominantly through the inhibition of NF-κB in various cellular models [59,60]. Costunolide and parthenolide inhibited LPS-induced NF-κB activation in RAW 264.7 cells, with IC_50_ values of 0 and 2.5 μM, respectively [61]. Shanmugam et al. reported that parthenolide inhibits NF-κB at concentrations of 4–5 μM in 22Rv1 prostate cancer cells and HUVEC cells, affecting genes involved in apoptosis and cell proliferation [62]. Meanwhile, Sheehan et al. reported that parthenolide can inhibit NF-κB binding to DNA at concentrations ranging from 30 nM to 10 µM [63]. However, a study evaluating 44 germacranes in TNF-R-stimulated Jurkat T cells found that NF-κB binding to DNA was inhibited within a concentration range of 5–300 μM [64]. The effects of erioflorin and its acetate at effective concentrations ranging from 10 to 20 µM are consistent with these findings, though they suggest that in DU-145 cells, erioflorin is a relatively less potent NF-κB inhibitor compared to parthenolide and costunolide. However, these differences in effective concentrations may be due to variations in the cell models, inflammatory stimuli, and experimental techniques used. Erioflorin has been reported to inhibit NF-κB activity at 5 μM in HEK-293 cells, reducing its activity to 55.7% in the presence of TNFα [25]. The results of this study reinforce its potential as an NF-κB inhibitor, although one whose efficacy may depend on cellular context. Since NF-κB plays a key role in resistance to castration or androgen receptor (AR) antagonists, its inhibition represents a relevant therapeutic approach in advanced prostate cancer. Notably, NF-κB/p65 can induce AR expression [65], and combination treatments with natural NF-κB and AR inhibitors have shown promising results in castration-resistant prostate cancer [62,66,67]. The inhibition of NF-κB by erioflorin and its acetate could represent a combined therapeutic strategy to be evaluated in preclinical models.

## 5. Conclusions

Erioflorin and its acetate induce cell death in the DU-145 and 22Rv1 advanced prostate cancer cell lines by increasing ROS levels and disrupting mitochondrial function. Erioflorin specifically enhances this effect by altering the balance between pro-apoptotic (BAX) and anti-apoptotic (BCL-2) proteins. Both germacranes significantly inhibit the NF-κB signaling pathway, a process facilitated by the pronounced increase in cellular ROS. This inhibition prevents the phosphorylation of IκBα in DU-145 cells, a model of advanced prostate cancer characterized by aberrant NF-κB activation, thereby contributing to apoptosis.

The results of this study suggest that erioflorin exhibits significant potential as a therapeutic agent for advanced prostate cancer. The observed inhibition of NF-κB activation by erioflorin and its acetate reinforces their potential as modulators of key signaling pathways involved in cancer progression, consistent with previous research on germacrane sesquiterpene lactones and their ability to affect NF-κB activity. This is known to play a critical role in cancer cell survival and inflammation, especially in the context of advanced prostate cancer. Nevertheless, despite the promising findings, further investigations, including in vivo studies, are necessary to validate these results and assess their clinical relevance.

## Data Availability

The data underlying this study are available in the article and its Supporting Information. FAIR Data (primary FID files for erioflorin and erioflorin acetate) are available at https://zenodo.org/records/14652072.

## Supplementary Materials

The following supporting information can be downloaded at: https://www.mdpi.com/article/10.3390/jox15020045/s1, Copies of 1H-NMR, 13C-NMR, COSY, HSQC, HMBC, and NOESY-spectra; NMR-data and NMR-signal assignments and comparison with reference data for erioflorin and erioflorin acetate (pdf). Figure S1. Title ^1^H NMR (500 MHz, CD_2_Cl_2_) of erioflorin. Figure S2. Title ^13^C NMR (125 MHz, CD_2_Cl_2_) of erioflorin. Figure S3. Title H,H-COSY (500 MHz, CD_2_Cl_2_) of erioflorin. Figure S4. Title HSQC (500/125 MHz, CD_2_Cl_2_) of erioflorin. Figure S5. Title HMBC (500/125 MHz, CD_2_Cl_2_) of erioflorin. Figure S6. Title NOESY (500 MHz, CD_2_Cl_2_) of erioflorin. Figure S7. Title ^1^H NMR (500 MHz, CD_2_Cl_2_) of erioflorin acetate. Figure S8. Title ^13^C NMR (125 MHz, CD_2_Cl_2_) of erioflorin acetate. Figure S9. Title H,H-COSY (500 MHz, CD_2_Cl_2_) of erioflorin acetate. Figure S10. Title HSQC (500/125 MHz, CD_2_Cl_2_) of erioflorin acetate. Figure S11. Title HMBC (500/125 MHz, CD_2_Cl_2_) of erioflorin acetate. Table S1. Title NMR-data of erioflorin and comparison with literature data. Table S2. Title NMR-data of erioflorin acetate and comparison with literature data.

## Abbreviations

The following abbreviations are used in this manuscript:

AP-1	Activator Protein-1
AR	Androgen receptor
BAX	BCL2 associated X, apoptosis regulator
BCL-2	BCL2 apoptosis regulator
DMSO	Dimethyl sulfoxide
EA	Erioflorin acetate
FBS	Fetal bovine serum
H2DCFDA	Dichlorodihydrofluorescein diacetate
IC50	Half maximal inhibitory concentration
LPS	Lipopolysaccharide
NF-κB	Nuclear transcription factor kappa B
Pdcd4	Programmed Cell Death Protein 4
ROS	Reactive oxygen species
SEAP	Secreted Embryonic Alkaline Phosphatase
TBS-T	Tris-buffered saline with Tween® 20
TMRM	Tetramethylrhodamine methyl ester
