# Supplementary material for: Erioflorin and Erioflorin Acetate Induce Cell Death in Advanced Prostate Cancer Through ROS Increase and NF-κB Inhibition
Source: J Xenobiot. 2025 Mar 18;15(2):45. doi: 10.3390/jox15020045 (PMC11932318; doi:10.3390/jox15020045)

Supporting Information

Erioflorin and Erioflorin acetate induce cell death in advanced prostate cancer through ROS increase and NF-κB inhibition

Cecilia Villegas [1], Iván González-Chavarría [2], Viviana Burgos [3], Jaime R. Cabrera-Pardo [4], Bernd Schmidt [5], and Cristian Paz [1]*

1. *Laboratory of Natural Products & Drug Discovery, Center CEBIM, Department of Basic Sciences, Faculty of Medicine, Universidad de La Frontera, Temuco 4780000, Chile. c.villegas04@ufromail.cl (C.V.)*
2. *Departamento de Fisiopatología, Facultad de Ciencias Biológicas, Universidad de Concepción, Concepción, Chile. ivancsbiologicas@gmail.com (I.G-C.)*
3. *Escuela de Tecnología Médica, Facultad de Salud, Universidad Santo Tomás, Chile; vburgos7@santotomas.cl (V.B.)*
4. *Laboratorio de Química Aplicada y Sustentable (LabQAS), Departamento de Química, Universidad del Bío-Bío, Avenida Collao 1202, Concepción 4051381, Chile; jacabrera777@gmail.com (J.R.C-P.)*
5. *Institut für Chemie, Universität Potsdam, Karl-Liebknecht-Str. 24-25, Potsdam D-14476, Germany; bernd.schmidt@uni-potsdam.de*

**Correspondence: cristian.paz@ufrontera.cl; Tel.: +56 45 259 2825.*

Contents:

NMR-Data assignment and comparison with literature data for erioflorin and erioflorin acetate; copies of 1D- and 2D-NMR spectra

A NMR-spectroscopical data and copies of spectra for erioflorin

Table S1. NMR-data of erioflorin and comparison with literature data.

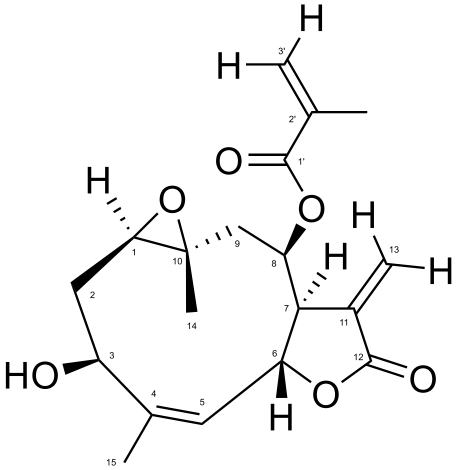

	This work[a]			Literature data for comparison[b]		
position	δ(^{1}H)/ppm	m (*J* (Hz))	δ(^{13}C)/ppm	δ(^{1}H)/ppm	m/*J* (Hz)	δ(^{13}C)/ppm
1	2.81	dd (10.2, 4.4)	60.8	2.79	dd (8.3, 2.7)	60.5
2	2.46<br>1.68	dt (14.9, 4.9)<br>ddd (14.9, 10.3, 2.5)	33.2	2.45<br>1.73	dt (12.4, 3.7)<br>ddd (12.4, 8.5, 2.0)	32.6
3	4.49	m	72.7	4.48	dd (3.7, 2.0)	71.9
4	--	--	142.1	--	--	141.6
5	5.31	dq (10.9, 1.5)	126.6	5.31	dq (9.1, 1.0)	125.9
6	6.64	dd (10.9, 2.2)	74.6	6.65	dd (9.1, 1.6)	74.1
7	2.90	m	48.8	2.87	m	48.2
8	5.15	m	77.1	5.16	m	76.5
9	2.77<br>1.33	dd (15.1, 4.8)<br>dd (15.1, 2.7)	43.9	2.81<br>1.32	dd (12.6, 3.7)<br>dd (12.6, 1.6)	43.4
10	--	--	58.6	--	--	58.6
11	--	--	138.0	--	--	137.1
12	--	--	169.8	--	--	169.3
13	5.77<br>6.29	d (2.1)<br>d (2.1)	124.8	5.75<br>6.35	d (1.5)<br>d (1.5)	124.6
14	1.43	s	20.0	1.44	s	19.6
15	1.81	d (1.4)	23.2	1.80	d (0.75)	22.8
1'	--	--	166.2	--	--	165.7
2'	--	--	136.1	--	--	135.1
3'	5.60<br>6.07	m<br>m	126.8	5.59<br>6.09	s(br)<br>s(br)	126.6
2'-CH_3	1.90	m	18.3	1.90	s	18.0

[a] ^{1}H NMR (500 MHz, CD_2Cl_2); ^{13}C NMR (125 MHz, CD_2Cl_2). [b] ^{1}H NMR (600 MHz, $CDCl_3$): Blees, J. S.; Bokesch, H. R.; Rübsamen, D.; Schulz, K.; Milke, L.; Bajer, M. M.; Gustafson, K. R.; Henrich, C. J.; McMahon, J. B.; Colburn, N. H.; Schmid, T.; Brüne, B. Erioflorin Stabilizes the Tumor Suppressor Pdcd4 by Inhibiting Its Interaction with the E3-ligase β-TrCP1. *PLOS ONE* **2012**, *7*, e46567; ^{13}C NMR (frequency not reported, $CDCl_3$): Morimoto, H.; Oshio, H. Isolation of Deacetylviguiestenin and Erioflorin From *Helianthus tuberosus*. *J. Nat. Prod.* **1981**, *44*, 748-749.

Figure S1. ^{1}H NMR (500 MHz, CD_2Cl_2) of erioflorin

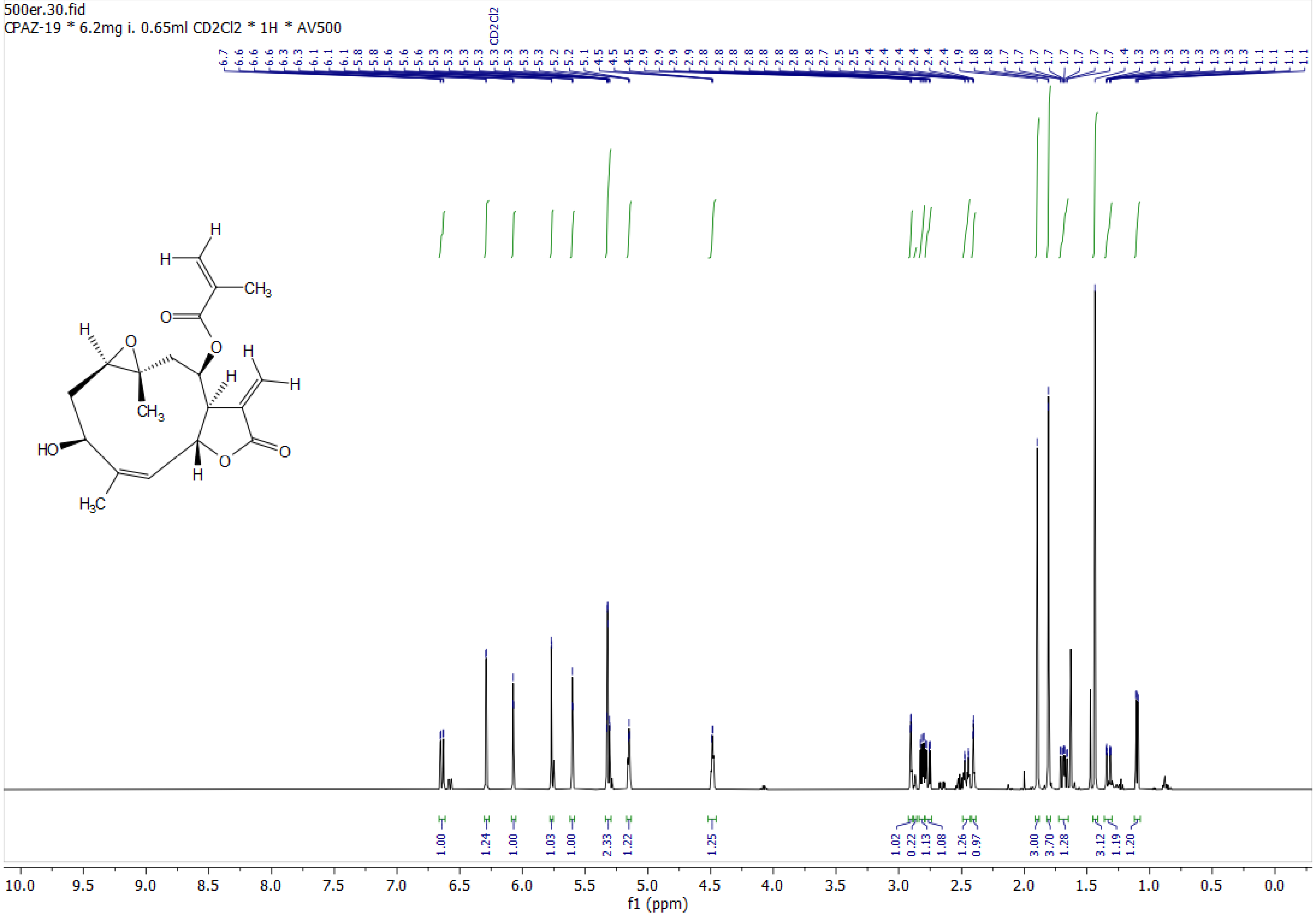

Figure S2. ^{13}C NMR (125 MHz, CD_2Cl_2) of erioflorin

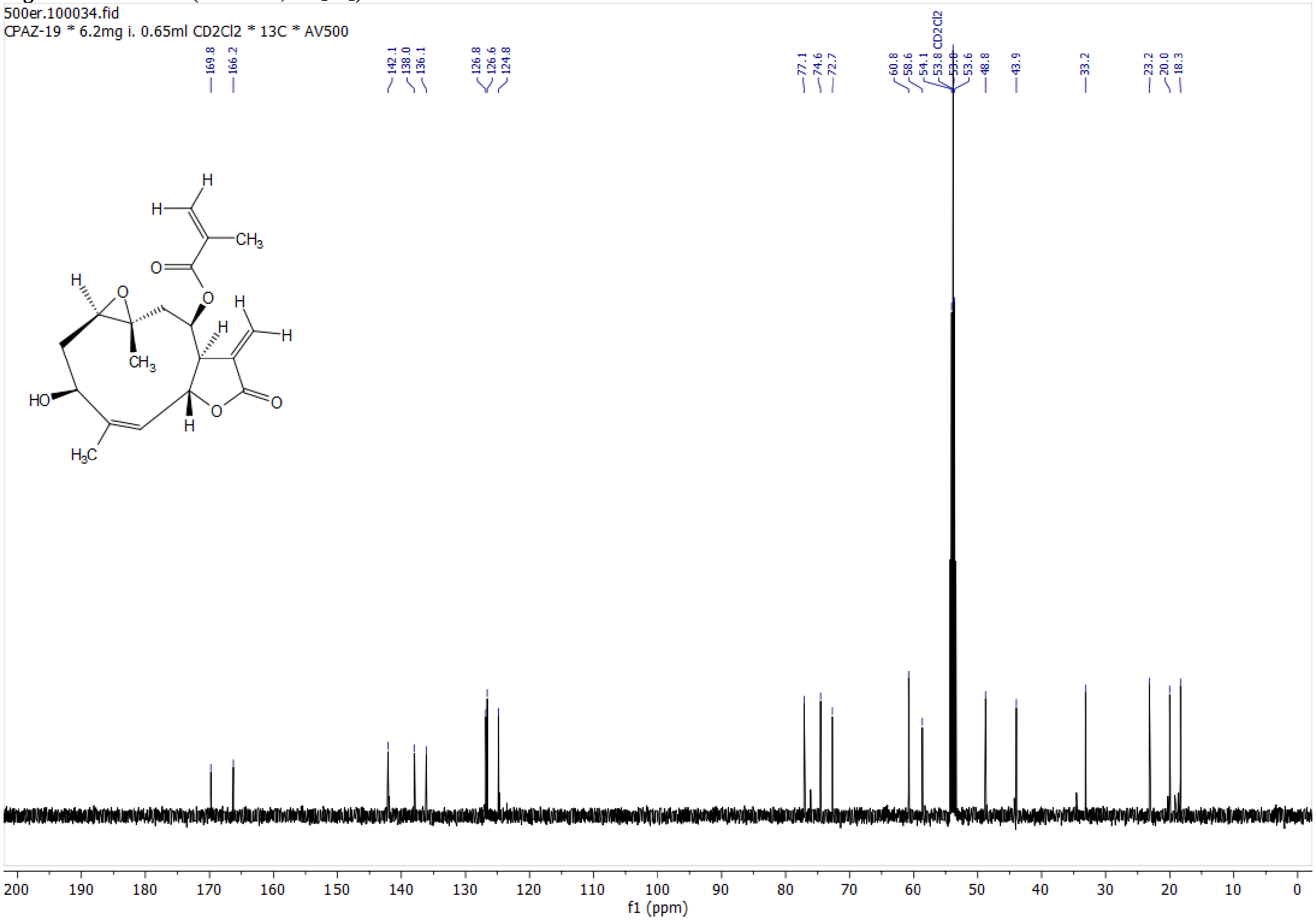

Figure S3: H,H-COSY (500 MHz, CD_2Cl_2) of erioflorin

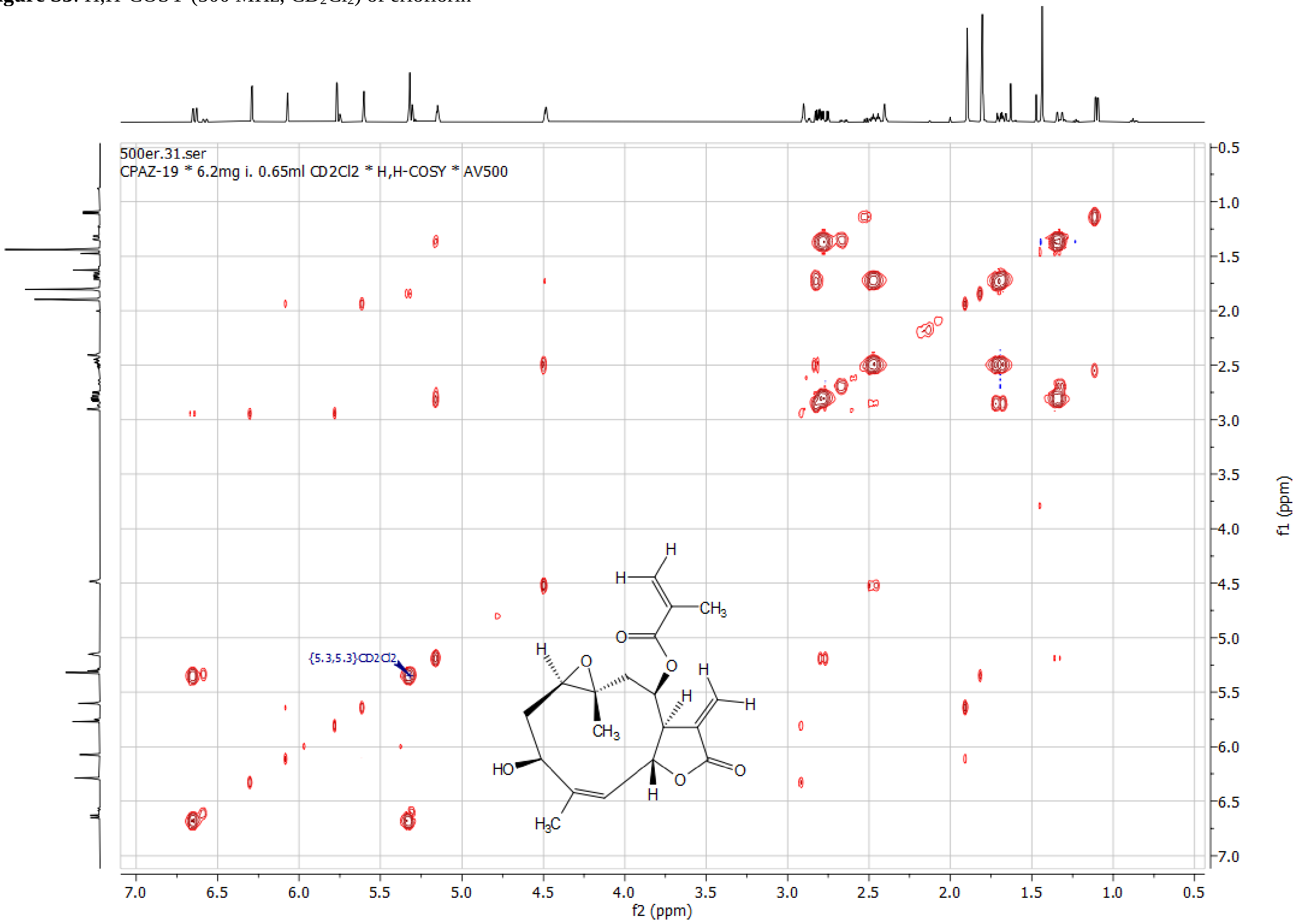

Figure S4: HSQC (500/125 MHz, CD_2Cl_2) of erioflorin

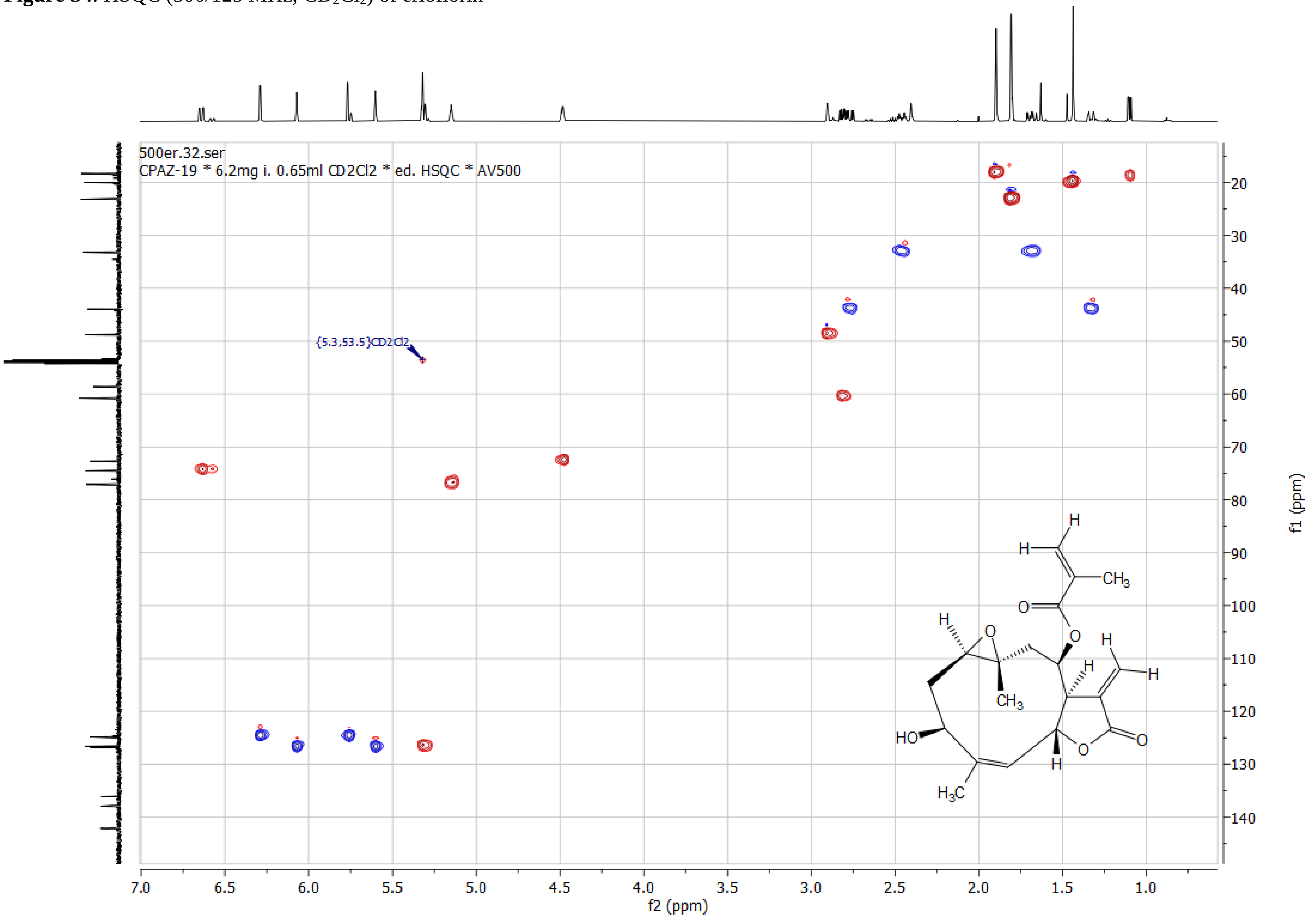

Figure S5: HMBC (500/125 MHz, CD_2Cl_2) of erioflorin

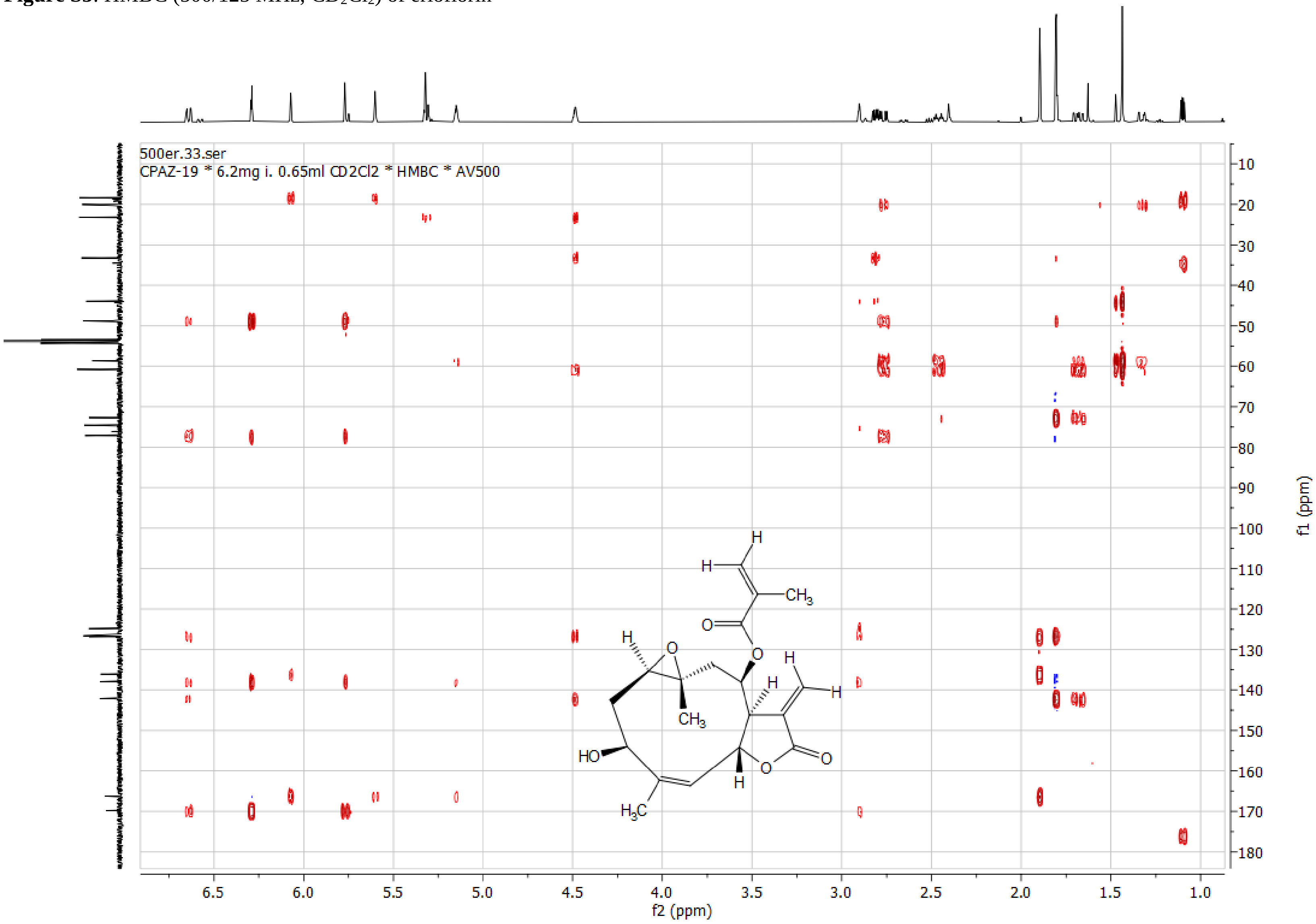

Figure S6: NOESY (500 MHz, CD_2Cl_2) of erioflorin

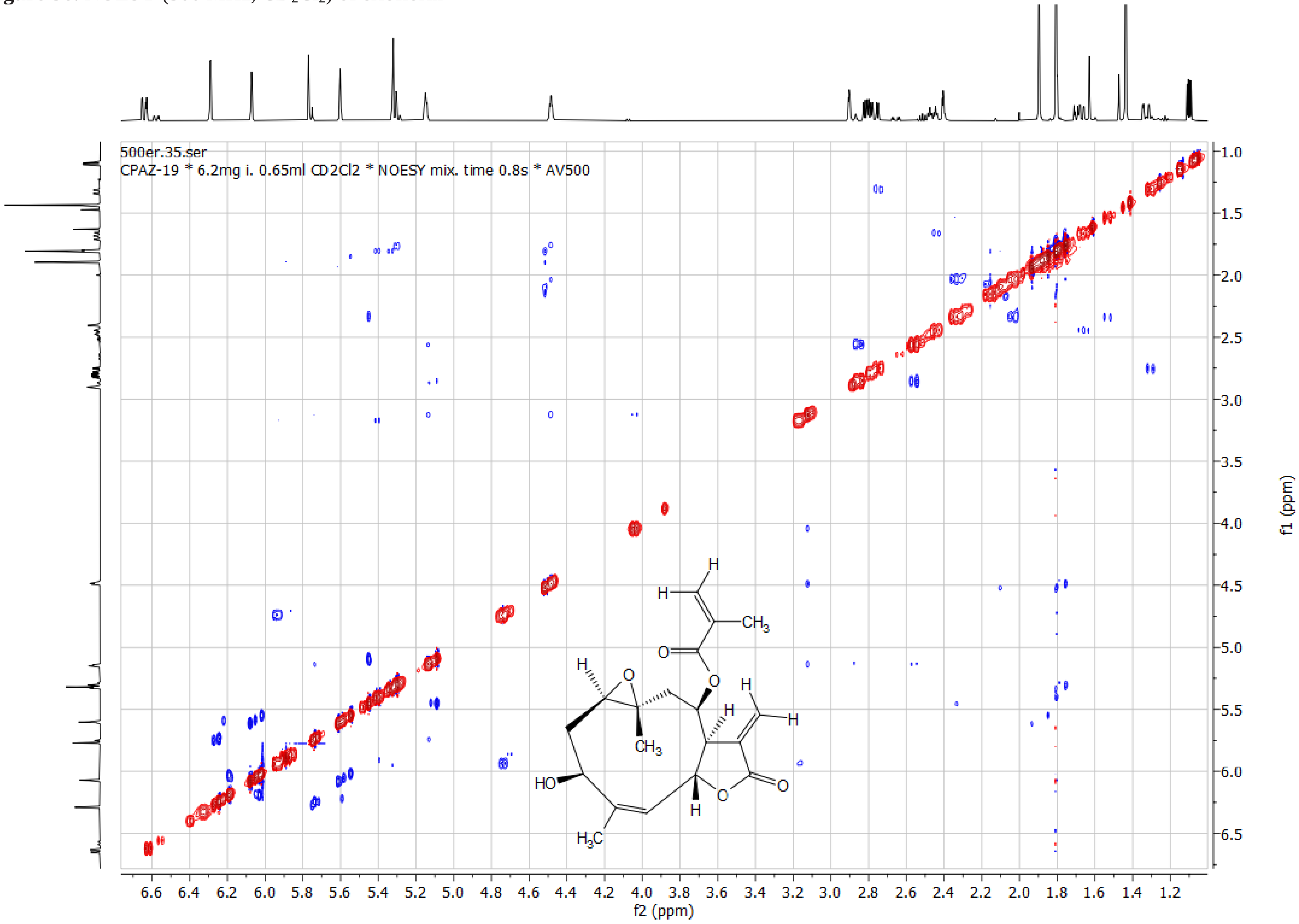

B NMR-spectroscopical data and copies of spectra for erioflorin acetate

Table S2. NMR-data of erioflorin acetate and comparison with literature data.

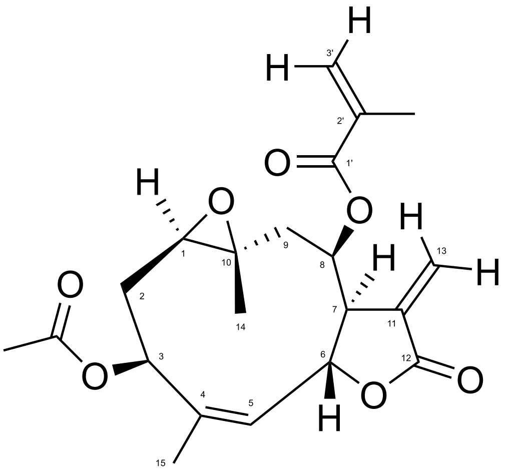

	This work[a]			Literature data for comparison[b]		
position	δ(^{1}H)/ppm	m (*J* (Hz))	δ(^{13}C)/ppm	δ(^{1}H)/ppm	m/*J* (Hz)	δ(^{13}C)/ppm
1	2.87	dd (10.2, 4.5)	60.5	2.62	dd (10, 4)	not reported
2	1.70<br>2.56	ddd (15.5, 10.2, 2.4)<br>dt (15.4, 4.6)	30.8	1.75<br>2.47	m<br>m	not reported
3	5.22	dd (4.9, 2.3)	73.5	ca. 5.2	m	not reported
4	--	--	138.9	--		not reported
5	5.28	dq (11.2, 1.6)	126.1	ca. 5.2	m	not reported
6	6.13	dd (11.2, 2.1)	74.8	6.11	dd (11, 2)	not reported
7	2.91	m	48.6	ca. 2.8	m	not reported
8	5.16	ddd (4.6, 2.6, 1.3)	77.0	ca. 5.2	m	not reported
9	1.35<br>2.80	dd (15.1, 2.4)<br>dd (15.1, 4.6)		ca. 2.8<br>1.4	m<br>dd (15, 2)	not reported
10	--	--	58.4	--	--	not reported
11	--	--	137.4	--	--	not reported
12	--	--	169.5	--	--	not reported
13	6.30<br>5.78	d (2.1)<br>d (2.1)		5.68<br>6.32	d (2.0)<br>d (2.0)	not reported
14	1.45	s	19.7	1.45	s	not reported
15	1.89	d (1.5)	23.1	1.88	d (2.0)	not reported
1'	--	--	166.0	--	--	not reported
2'	--	--	136.2	--	--	not reported
3'	6.08<br>5.62	m<br>m	126.8	not reported		not reported
2'-CH_3	1.92	m	18.3	not reported		not reported
O*C*(O)CH_3	--	--	169.5	--	--	not reported
OC(O)*CH_3*	2.10	s	21.3	2.1	s	not reported

[a] ^{1}H NMR (500 MHz, CD_2Cl_2); ^{13}C NMR (125 MHz, CD_2Cl_2). [b] ^{1}H NMR (100 MHz, $CDCl_3$): original NMR chemical shift values were reported in τ and have been recalculated to δ values for this comparison (δ = 10 – τ): Gnecco, S.; Poyser, J. P.; Silva, M.; Sammes, P. G.; Tyler, T. W. Sesquiterpene lactones from *Podanthus ovatifolius*. *Phytochemistry* **1973**, *12*, 2469-2477. No ^{13}C NMR data have been reported in the literature for erioflorin acetate before.

Figure S7. ^{1}H NMR (500 MHz, CD_2Cl_2) of erioflorin acetate

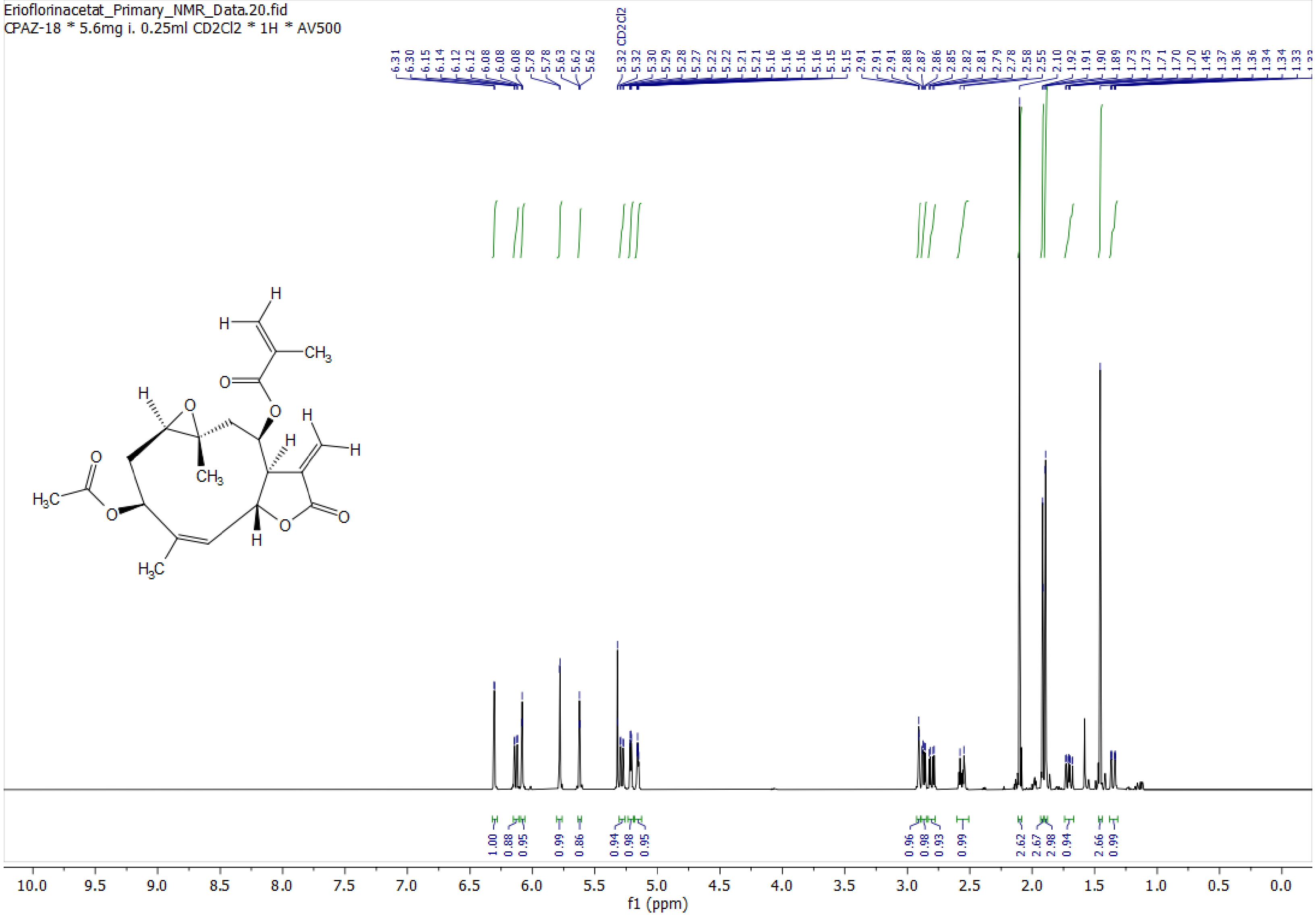

Figure S8. ^{13}C NMR (125 MHz, CD_2Cl_2) of erioflorin acetate

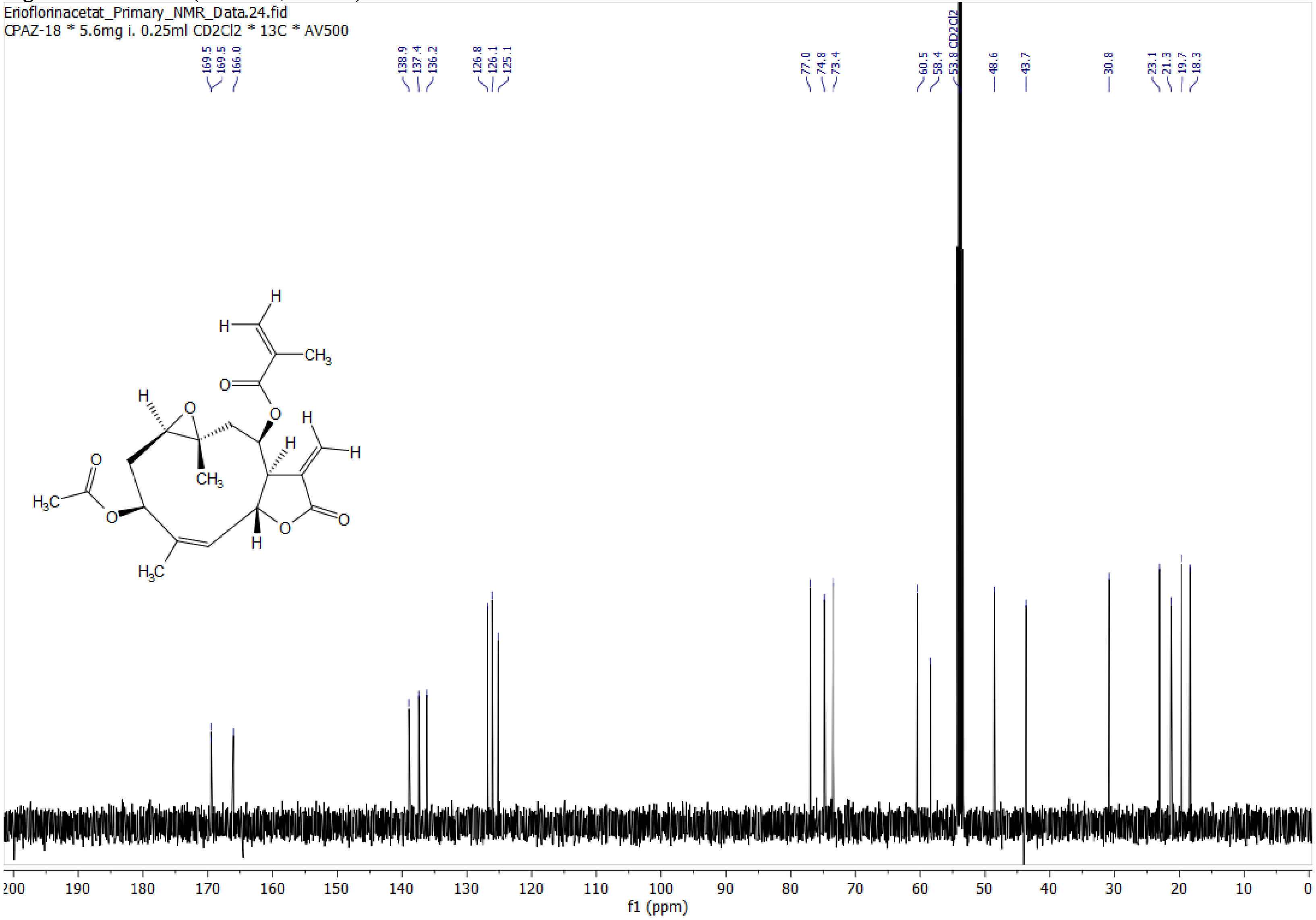

Figure S9: H,H-COSY (500 MHz, CD_2Cl_2) of erioflorin acetate

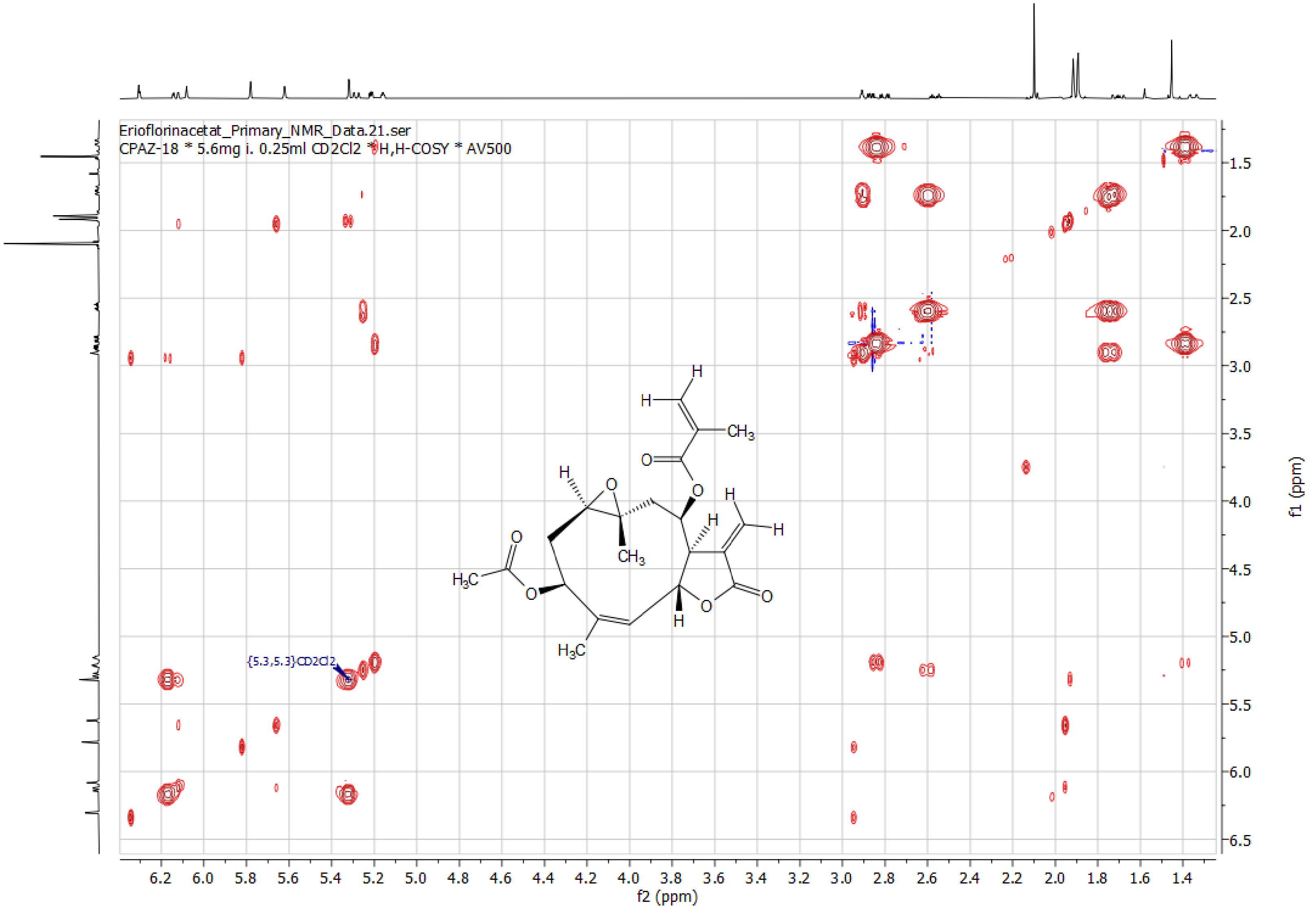

Figure S10: HSQC (500/125 MHz, CD_2Cl_2) of erioflorin acetate

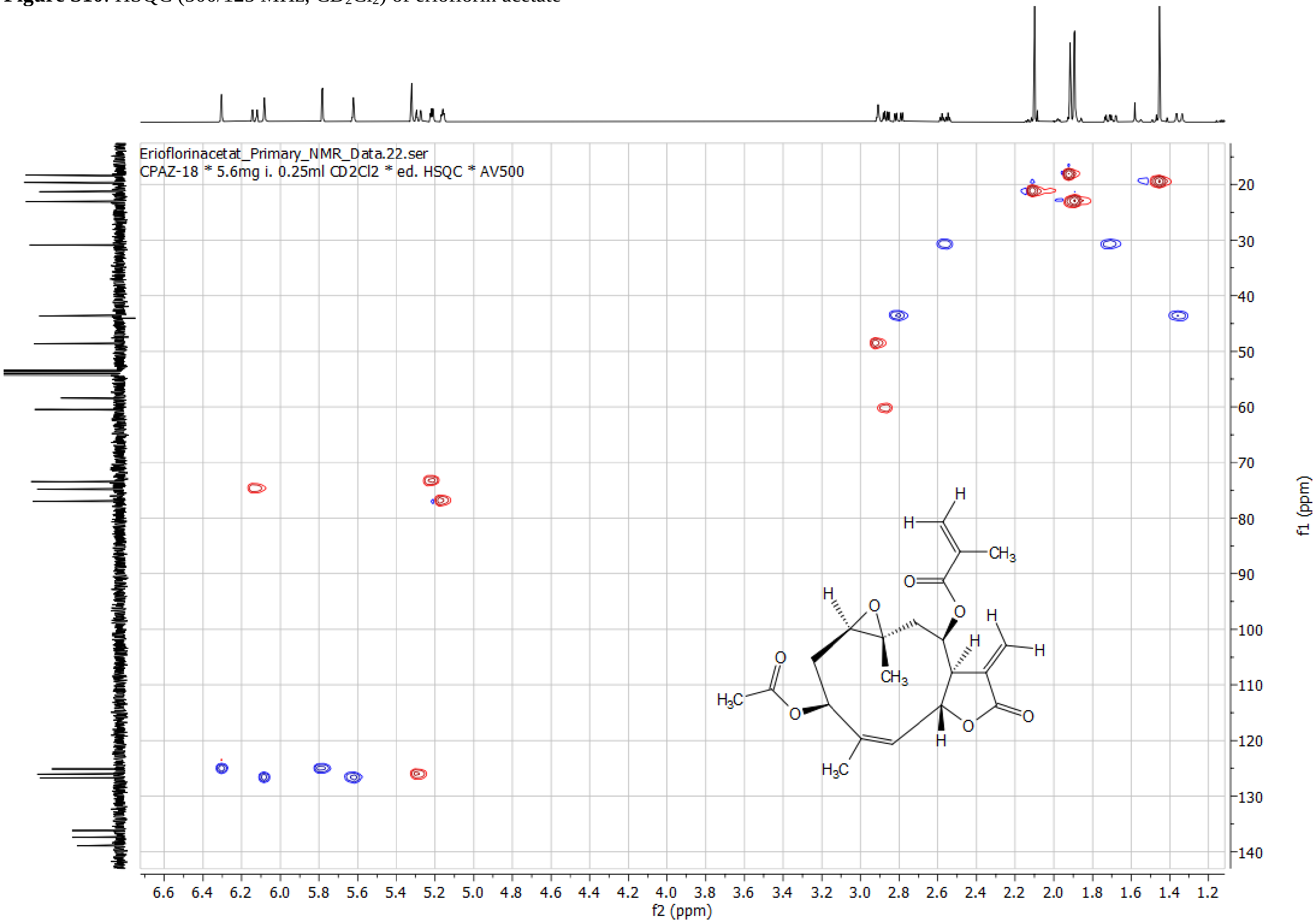

Figure S11: HMBC (500/125 MHz, CD_2Cl_2) of erioflorin acetate

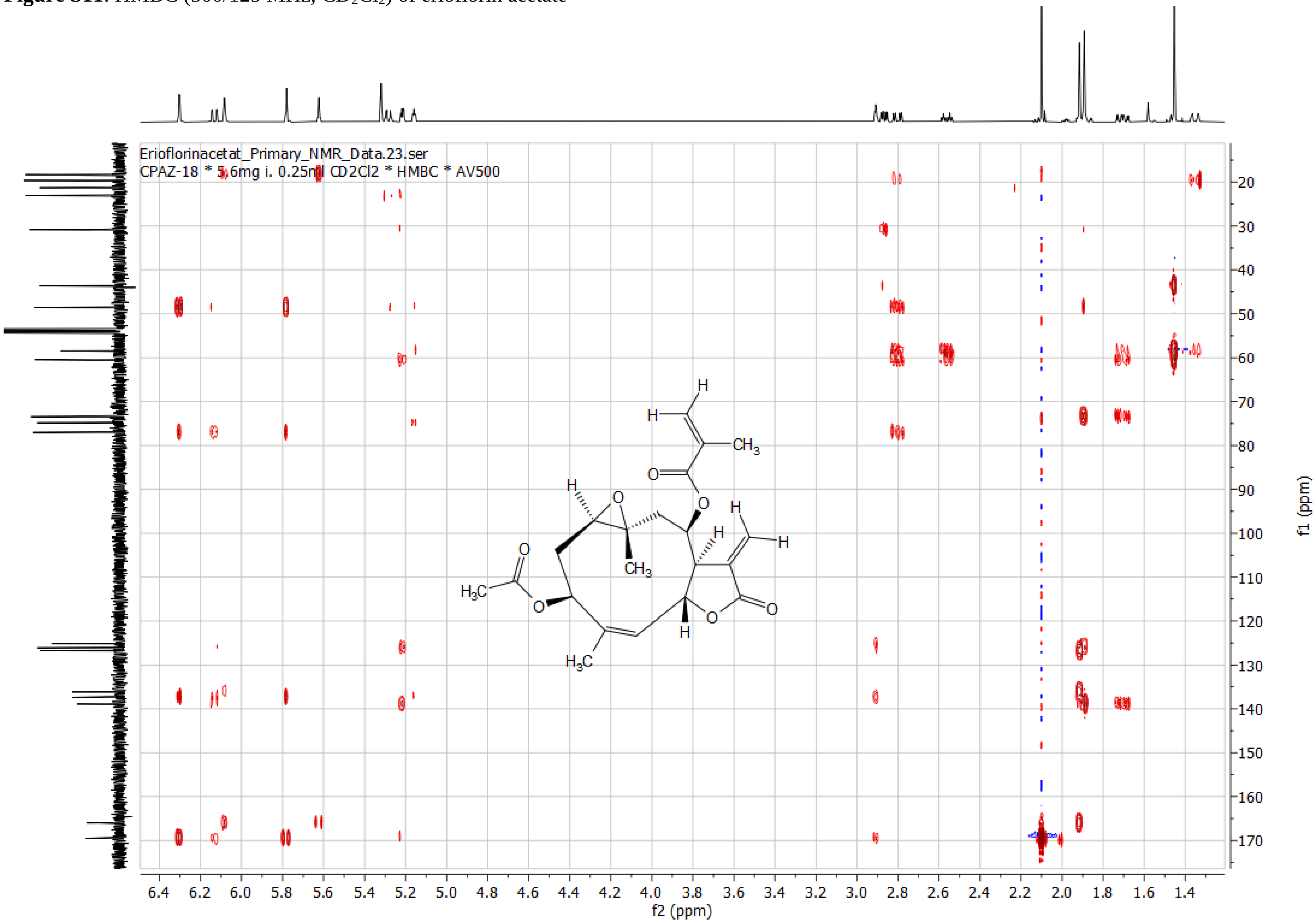

Supplement: Supplementary file 1 [file jox-15-00045-s001.zip › jox-3469324-supplementary.pdf]
